# Higher matrix stiffness as an independent initiator triggers epithelial-mesenchymal transition and facilitates HCC metastasis

**DOI:** 10.1186/s13045-019-0795-5

**Published:** 2019-11-08

**Authors:** Yinying Dong, Qiongdan Zheng, Zhiming Wang, Xiahui Lin, Yang You, Sifan Wu, Yaohui Wang, Chao Hu, Xiaoying Xie, Jie Chen, Dongmei Gao, Yan Zhao, Weizhong Wu, Yinkun Liu, Zhenggang Ren, Rongxin Chen, Jiefeng Cui

**Affiliations:** 1 Liver Cancer Institute, Zhongshan Hospital, Fudan University & Key Laboratory of Carcinogenesis and Cancer Invasion, Ministry of Education, 136 Yi Xue Yuan Road, Shanghai, 200032 People’s Republic of China; 2 Department of Oncology, Zhongshan Hospital, Fudan University, Shanghai, 200032 People’s Republic of China; 3 Department of Radiology, Shanghai Cancer Center, Fudan University, Shanghai, 200032 People’s Republic of China; 4 Department of Urology, Zhongshan Hospital, Fudan University, Shanghai, 200032 People’s Republic of China

**Keywords:** Hepatocellular carcinoma, Matrix stiffness, S100A11, eIF4E, TGF β1

## Abstract

**Background:**

Increased liver stiffness exerts a detrimental role in driving hepatocellular carcinoma (HCC) malignancy and progression, and indicates a high risk of unfavorable outcomes. However, it remains largely unknown how liver matrix stiffness as an independent cue triggers epithelial-mesenchymal transition (EMT) and facilitates HCC metastasis.

**Methods:**

Buffalo rat HCC models with different liver stiffness backgrounds and an in vitro Col I-coated cell culture system with tunable stiffness were used in the study to explore the effects of matrix stiffness on EMT occurrence and its underlying molecular mechanism. Clinical significance of liver stiffness and key molecules required for stiffness-induced EMT were validated in HCC cohorts with different liver stiffness.

**Results:**

HCC xenografts grown in higher stiffness liver exhibited worse malignant phenotypes and higher lung metastasis rate, suggesting that higher liver stiffness promotes HCC invasion and metastasis. Cell tests in vitro showed that higher matrix stiffness was able to strikingly strengthen malignant phenotypes and independently induce EMT occurrence in HCC cells, and three signaling pathways converging on Snail expression participated in stiffness-mediated effect on EMT including integrin-mediated S100A11 membrane translocation, eIF4E phosphorylation, and TGF β1 autocrine. Additionally, the key molecules required for stiffness-induced EMT were highly expressed in tumor tissues of HCC patients with higher liver stiffness and correlated with poor tumor differentiation and higher recurrence.

**Conclusions:**

Higher matrix stiffness as an initiator triggers epithelial-mesenchymal transition (EMT) in HCC cells independently, and three signaling pathways converging on Snail expression contribute to this pathological process. This work highlights a significant role of biomechanical signal in triggering EMT and facilitating HCC invasion and metastasis.

## Background

Extracellular matrix proteins, as the most abundant noncellular solid-state component in tumor microenvironment, not only maintain three-dimensional morphological architecture of tumor cell/tissue, but also produce biochemical or biophysical signals to influence biological functions of tumor cells. Currently, the biochemical effects of matrix proteins on invasion and metastasis of tumor cells have been well documented [1–3], but their biophysical signal effects are less explored. Matrix stiffening is the most remarkable mechanical and physical feature of solid tumor, which mainly results from the excessive deposition and crosslinking of extracellular matrix proteins [4, 5]. This mechanical stiffness may break the balance of cellular surface force, promote integrin clustering and focal adhesion formation which transmit exogenous matrix force signals into cells, and ultimately affect their biological phenotypes and characteristics such as cell morphology, cell growth, and differentiation, as well as the synthesis/secretion of proteins/cytokines and metabolism [6–12]. Additionally, it also modulates cell migration through cytoskeleton remodeling [13]. Clinical data have demonstrated that the majority of hepatocellular carcinoma (HCC) develop on the basis of the fibrotic or cirrhotic livers, and HCC patients with severe cirrhosis are inclined to have shorter median survival time [14, 15]. Higher liver stiffness facilitates HCC development and progression, also indicates a high risk of unfavorable outcomes [16, 17]. Therefore, liver matrix stiffness may act as a critical modulator to alter tumor behavior and progression.

Higher matrix stiffness promotes proliferation and chemotherapeutic resistance [18], upregulates VEGF and OPN expression [19, 20], enhances stemness [21] in HCC cells, also elevates integrin β1 expression in HCC tissue, and determines HCC malignancy [22]. Besides these malignant alterations, higher matrix stiffness-upregulated LOXL2 facilitates pre-metastatic niche formation in lung [23]. All these findings suggest a strong linkage between matrix stiffness and HCC invasion/metastasis. Nevertheless, it is still elusive whether and how matrix stiffness as an independent cue triggers epithelial-mesenchymal transition (EMT) and facilitates HCC metastasis.

EMT frequently occurs in the initiation of tumor invasion and metastasis. Soluble factors, ECM components, and hypoxia are able to induce a transition of tumor cells from epithelial state to mesenchymal state [24]. Except that, matrix stiffness in cooperation with soluble factors can also govern tumor cell undergoing a mesenchymal shift and in turn drive the progress of metastasis [25]. Higher matrix stiffness enhances TGF-β1-induced Smad signaling in HCC cells [18], controls TGF-β-induced EMT in mammalian gland cells and kidney epithelial cells [5], and regulates MMP3-induced EMT in mammalian epithelial cells [26]. However, little is known about how matrix stiffness alone as an independent cue initiates EMT in HCC cells. Considering that tumor cells undergoing EMT exhibit fibroblast-like morphology, cytoskeleton remodeling, protrusive and invasive pseudopodial structure, similar with morphology, and greater migration capability of tumor cells under high stiffness stimulation, we speculate that higher matrix stiffness may trigger EMT in HCC cells, and pseudopod-associated proteins may participate in matrix stiffness-mediated EMT.

In the study, using buffalo rat HCC models with different liver stiffness backgrounds and an in vitro Col I-coated cell culture system with tunable stiffness, we clarified the underlying molecular mechanisms by which matrix stiffness alone as an initiator induced EMT occurrence in HCC. This work highlights a significant role of biomechanical signal in triggering EMT and facilitating HCC invasion and metastasis, and opens a promising therapeutics approach targeting on matrix stiffness for prevention of HCC metastasis.

## Methods

### Establishment of buffalo rat HCC models with different liver stiffness backgrounds

Thirty-six buffalo rats (6-week-old, Charles River Laboratories, USA) were randomly divided into 3 groups. The rats in group M were intraperitoneally injected with 100% CCl_4_ (3 ml/kg) followed by 50% CCl_4_ olive solution (2 ml/kg) once a week for 12 weeks, the rats in group H with 100% CCl_4_ (3 ml/kg) followed by 50% CCl_4_ olive solution (2 ml/kg) twice a week for 12 weeks, and the rats in group N with saline for 12 weeks. Live stiffness of the rats in 3 groups (6 rats each group) were measured using a TA.Xtplus Texture Analyzer (Stable Micro Systems Ltd., UK). Subsequently, a subcutaneous tumor tissue mass (2 × 2 × 2 mm^3^) derived from McA-RH7777 cells was orthotopically transplanted into livers of other 18 rats in 3 groups (6 rats each group). Twenty-five days later, HCC tumor-bearing rats with different liver stiffness backgrounds were formed. All animal care and experiments were in accordance with the guideline for the Care and Use of Laboratory Animals published by the National Academy of Science, and the related experiment design met with approval from the Animal Care Ethical Committee of Fudan University.

### Subcutaneous tumorigenicity analysis of HCC cells mixed in Matrigel with various concentration Col1

Twenty-four 4-week-old male BALB/c nude mice were obtained from Shanghai SLAC Laboratory Animal Co., Shanghai, China. Appropriate 1 × 10^7^ MHCC97H cells or 1.5 × 10^7^ Hep3B cells were suspended in C solution (100 μl serum-free medium), Matr solution (6 mg/ml Matrigel in 100 μl serum-free medium), Matr+L solution (6 mg/ml Matrigel and 3.5 mg/ml COL1 in 100μlserum-free medium), and Matr+H solution (6 mg/ml Matrigel and 70 mg/ml COL1 in 100 μl serum-free medium), respectively. The suspended HCC cells were subcutaneously injected into the upper left flank region of nude mice, and the growth of subcutaneous tumors was observed.

### Preparation of in vitro system of mechanically tunable COL1-coated polyacrylamide gel

An in vitro system of mechanically tunable COL1-coated polyacrylamide gels was done as our previous study [19]. For details, please see Additional file 1: Supporting Information.

### Patients and HCC tissues

We retrospectively collected the clinical data of 74 HCC patients who received preoperative assessments for liver stiffness by two-dimensional shear wave elastography and underwent curative resection in the Zhongshan Hospital of Fudan University (Shanghai, China) during July 2015 to August 2017. Patients did not receive any preoperative anticancer therapy. We classified these patients into three groups according to the values of liver stiffness: group I (≤ 8 kPa), group II (> 8 and < 12 kPa), and group III (≥ 12 kPa). Thirty HCC tissue specimens of 74 patients (10 tissue samples each group) were obtained from tissue bank. Tumor differentiation was assessed according to the Edmondson grading system. Histological grading of inflammation (G0–G4) and staging of fibrosis (S0–S4) for non-tumoral liver were evaluated according to the Metavir scoring criteria. All the patients after surgical resection were followed up to October 2018, with a median follow-up of 30.2 months (range 3.6–38.5 months). The study was approved by the Zhongshan Hospital Research Ethics Committee, and written informed consent obtained from each patient.

### Statistics

Statistical analyses were performed using SPSS 23.0 (SPSS Inc., Chicago, IL, USA). Continuous variables were expressed as the mean ± standard deviation (SD), and categorical data were displayed as absolute number (*n*). The chi-squared test and rank sum test were used to compare qualitative variables, and quantitative variables were analyzed by the analysis of variance (ANOVA) test among three groups and Student's *t* test between two groups. A *P* < 0.05 (two-tailed) was considered statistically significant.

### Other materials and methods

For details of other materials and methods, please see Additional file 1: Supporting Information and Additional file 7: Table S1.

## Results

### Higher liver stiffness promotes HCC growth and metastasis in vivo

**Fig. 1:**
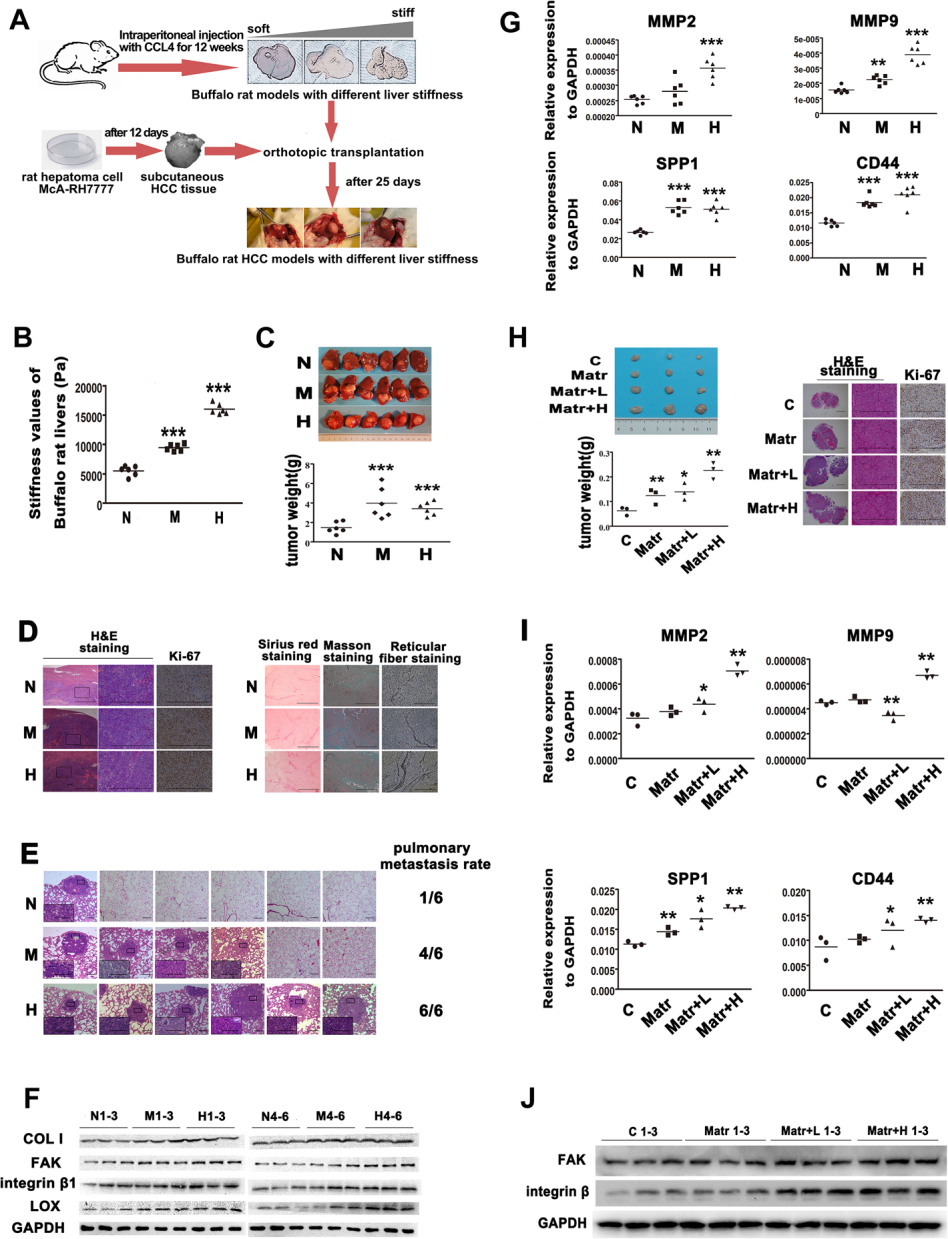
Higher liver stiffness promotes the growth and metastasis of HCC in vivo. **a** The flow chart for establishment of buffalo rat HCC models with different liver stiffness backgrounds. **b** Liver stiffness values of buffalo rats in groups N, M, and H. N, normal stiffness; M, medium stiffness; H, high stiffness. **c** Gross appearance and size of orthotopic HCC tumors in groups N, M, and H. **d** Histochemistry analysis of Sirius red, Masson’s trichrome, reticular fiber staining, and Ki-67 expression in orthotopic HCC tumors. **e** Pulmonary metastasis rate of orthotopic HCC tumors in groups N, M, and H. The incidences of lung metastasis in group M (4/6, 66.7%) and group H (6/6, 100%) were more frequent than that in groups N (1/6, 16.7%). **f** The expressions of COL1, FAK, integrin β1, and LOX in orthotopic HCC tumors in groups N, M, and H. **g** The expressions of MMP2, MMP9, SPP1, and CD44 in orthotopic HCC tumors in groups N, M, and H. **h** Gross appearance and wet weight of subcutaneous tumors derived from MHCC97H cells mixed in Matrigel and varied concentration of COL1, and their histochemistry analysis and Ki-67 expression. C, HCC cells; Matr, HCC cells in Matrigel; Matr+L, HCC cells in Matrigel and low concentration COL1; Matr+H, HCC cells in Matrigel and high concentration COL1. **i** The expressions of MMP2, MMP9, SPP1, and CD44 in subcutaneous tumors. **j** The expressions of integrin β1 and FAK in subcutaneous tumors. Scale bar, 1000 μm. Error bars indicate SD. *P < 0.05, **P < 0.01, ***P < 0.001

A flow chart for establishment of buffalo rat HCC models with different liver stiffness backgrounds was shown in Fig. 1a. Average liver stiffness of rats in the medium (M)- and high (H)-stiffness groups was significantly higher than that in the normal (N)-stiffness group (9.40 ± 0.54 kPa and 16.05 ± 0.96 kPa vs. 5.48 ± 0.85 kPa) (Fig. 1b). Liver stiffness ranges of three groups exactly mirrored the stiffness ranges of normal, fibrotic, and cirrhotic liver, respectively [27]. HCC xenografts grew faster in higher stiffness liver, and their tumor weight and Ki-67 expression all markedly increased (Fig. 1c, d), suggesting that higher liver stiffness promotes the growth of HCC. The incidences of lung metastasis in group M (4/6, 66.7%) and group H (6/6, 100%) were more frequent than that in group N (1/6, 16.7%) (Fig. 1e), demonstrating that higher liver stiffness facilitates HCC metastasis. Compared with the controls in group N, HCC tumors in groups M and H presented more collagenous fiber and reticular fiber deposition (Fig. 1d), higher expressions of LOX and Collagen I (Fig. 1f, Additional file 2: Figure S1A), and poorer liver function (Additional file 2: Figure S1B). Simultaneously, they also had significant increases in the expressions of stiffness-sensor molecules (integrin β1, FAK) and metastasis-associated genes (MMP2, MMP9, CD44, and SPP1) (Fig. 1f, g), implying that high stiffness signal is transduced into HCC cells to strengthen their metastatic potential. All the above results support that higher liver matrix stiffness promotes the growth and metastasis of HCC.

We further used a mixture of Matrigel with varied concentration of Collagen 1 to simulate different matrix stiffness for analyzing their effects on HCC tumorigenicity. Subcutaneous tumors in the group with Matrigel and high amount of COL1 grew faster and exhibited a remarkable upregulation in Ki-67 expression (Fig. 1h, Additional file 2: Figure S1C, D). Metastasis-associated gene (MMP2, MMP9, CD44, SPP1) and stiffness-sensor molecule (Integrin β1, FAK) expressions also showed an increasing inclination (Fig. 1i, j, Additional file 2: Figure S1E, F). These results were in agreement with the findings in rat HCC model described above and indicate that higher matrix stiffness enhances the growth and metastatic potential of HCC cells.

### Higher matrix stiffness alters malignant phenotypes and induces EMT occurrence in HCC cells independently

**Fig. 2:**
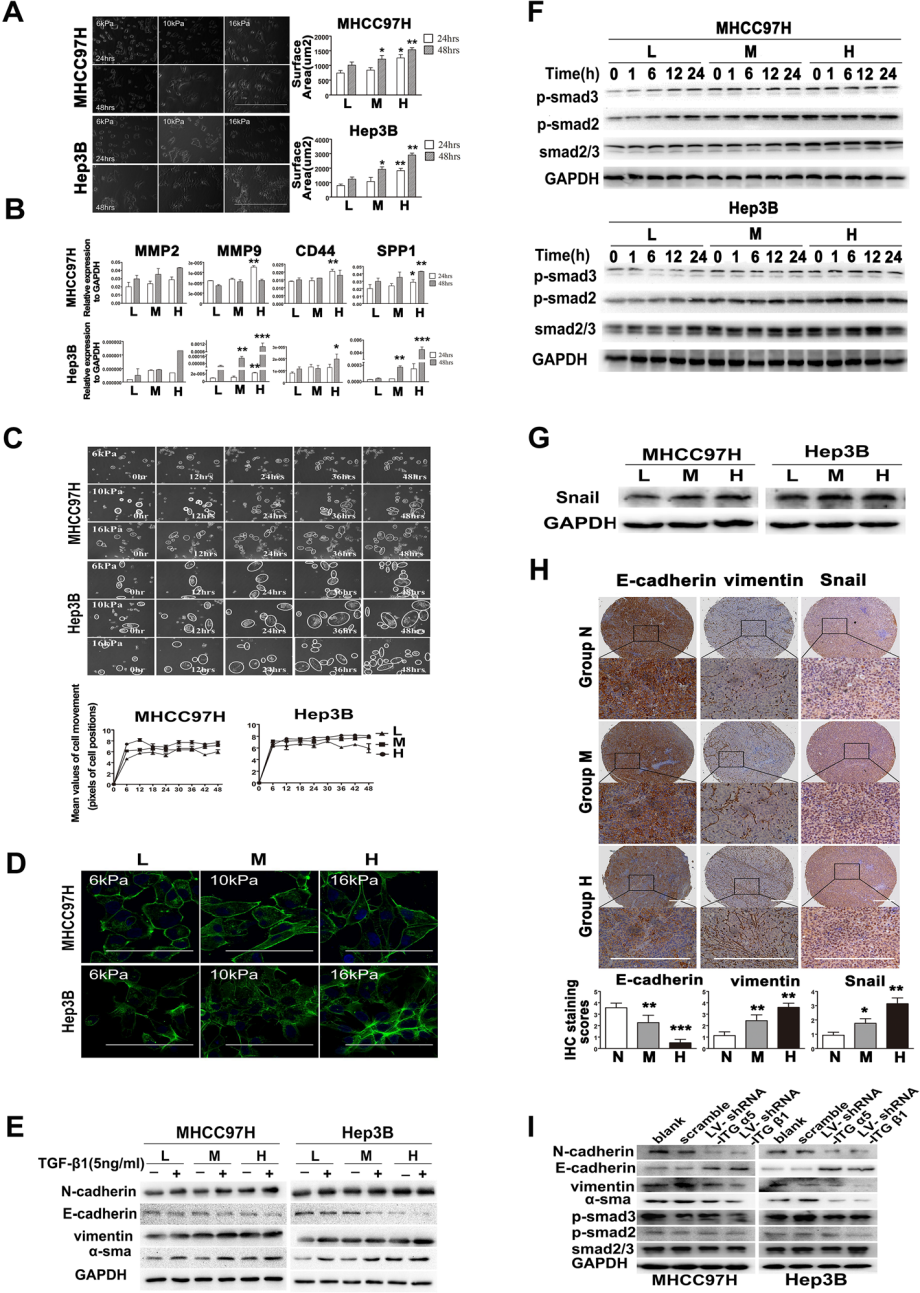
Higher matrix stiffness alters malignant phenotypes and induces EMT occurrence in HCC cells independently. **a** Alteration in appearance and spreading area of HCC cells under higher stiffness stimulation. L, low-stiffness substrate, 6 KPa; M, medium-stiffness substrate, 10 KPa; H, high-stiffness substrate, 16 KPa. Scale bar, 1000 μm. **b** The mRNA expressions of MMP2, MMP9, SPP1, and CD44 in HCC cells grown on different stiffness substrates detected by qRT-PCR. **c** Analysis of cell movement under different stiffness stimulations by the real-time cell monitoring system. **d** Alexa-488 Phalloidin staining showed that higher matrix stiffness promotes F-actin polymerization in HCC cells (scale bar, 350 μm). **e** The expressions of N-cadherin, vimentin, α-SMA, and E-cadherin in HCC cells grown on different stiffness substrates in presence or absence of TGF β1. **f** Phosphorylation levels of Smad2 and Smad3 at different time points of TGFβ1 stimulation. **g** Snail expression in HCC cells grown on different stiffness substrates. **h** Expressions of E-cadherin, vimentin, and Snail in orthotopic HCC tumors in groups N, M, and H. N, normal stiffness; M, medium stiffness; H, high stiffness. Scale bar, 1000 μm. **i** Suppression of integrin β1 or α5 reversed the expressions of EMT markers in HCC cells grown on higher stiffness substrate. LV-shRNA-ITGβ1, lentivirus-shRNA-integrinβ1; LV-shRNA-ITGα5, lentivirus-shRNA-integrinα5. *P < 0.05, **P < 0.01, ***P < 0.001

We established an in vitro Col I-coated culture system with tunable stiffness (Additional file 3: Figure S2A, B) and used 16 kPa, 10 kPa, and 6 kPa stiffness substrate to simulate the stiffness of cirrhotic, fibrotic, and normal liver for exploring matrix stiffness-mediated effects on HCC cells. HCC cells under higher stiffness stimulation exhibited a fibroblast-like morphology (Fig. 2a), higher expressions in metastasis-associated genes (Fig. 2b), and greater motility (Fig. 2c, d), suggesting that matrix stiffness may strengthen invasion and metastasis of HCC cells. These findings were consistent with the above results in vivo. Additionally, HCC cells grown on FN-coated, LN-coated, or COL1-coated substrates with tunable stiffness exhibited similar changes in morphology and expression pattern in Snail (Additional file 3: Figure S2C), implying that matrix stiffness stimulation but no matrix proteins is responsible for these changes in malignant phenotypes.

Given that stiffness-induced malignant phenotypes resembled as that of tumor cells undergoing EMT, we speculated that higher matrix stiffness might trigger EMT and facilitate HCC metastasis. Irrespective of the presence or absence of TGF β1, HCC cells under higher stiffness stimulation all showed an obvious decrease in E-cadherin expression and an increase in N-cadherin, vimentin, and α-SMA expression (Fig. 2e). Simultaneously, their Smad2/Smad3 phosphorylation level and Snail expression (Fig. 2f, g) also increased significantly. Consistently, HCC tissues in higher stiffness group demonstrated a significant downregulation in E-cadherin and a remarkable upregulation in vimentin and Snail, compared with those in normal stiffness group (Fig. 2h). Integrin β1 and integrin α5 as leading differential integrin subunits may deliver stiffness signals into HCC cells [19]. Knockdown of these two integrin subunits significantly suppressed the expressions of N-cadherin, vimentin, α-SMA, p-Smad2, and p-Smad3 in HCC cells grown on higher stiffness substrate, but upregulated E-cadherin expression (Fig. 2i, Additional file 3: Figure S2D). Taken all together, higher matrix stiffness alone was adequate to drive EMT in HCC cells via integrin β1 or integrin α5. Additionally, in the presence of exogenous TGF β1, HCC cells grown on higher stiffness substrate presented more complete EMT indicating a synergistic effect of matrix stiffness and TGFβ1 on EMT (Fig. 2e, f).

### Membrane translocation of S100A11 participates in higher stiffness-induced EMT in HCC cells

**Fig. 3:**
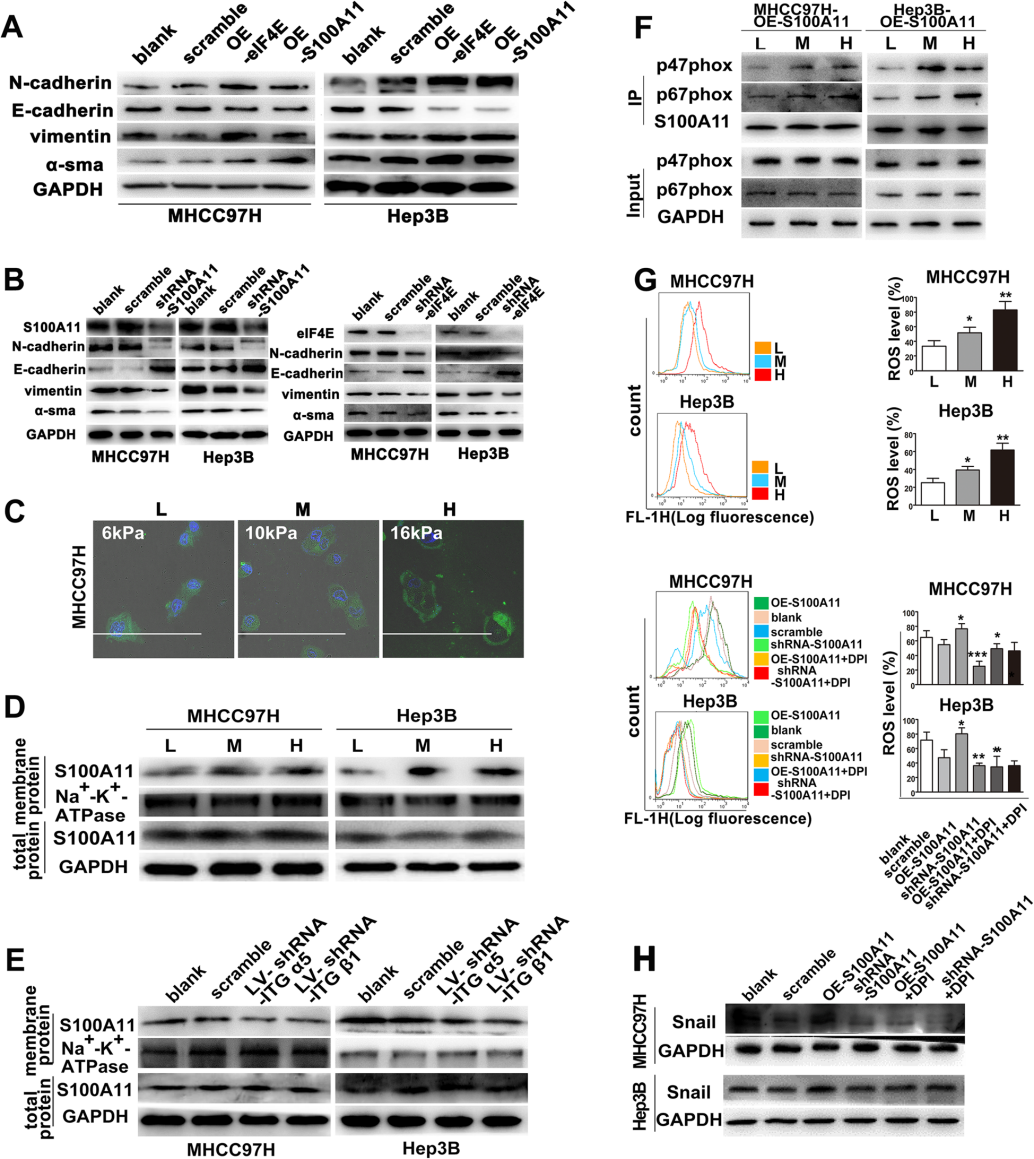
S100A11 membrane translocation participates in stiffness-induced mesenchymal shift in HCC cells. **a** Overexpression of S100A11 or eIF4E promoted an increase in N-cadherin, vimentin, and α-SMA expression and a decrease in E-cadherin expression in HCC cells grown on high stiffness substrate. **b** Knockdown of S100A11 or eIF4E partially attenuated high stiffness-induced EMT in HCC cells. **c** S100A11 distribution in HCC cells transfected with pEGFP-OE-S100A11 grown on different stiffness substrates. L, low-stiffness substrate, 6 KPa; M, medium-stiffness substrate, 10 KPa; H, high-stiffness substrate, 16 KPa. **d** S100A11 expression at membrane protein and total protein level in HCC cells under different stiffness stimulations. **e** Knockdown of integrin α5 or integrin β1 partially inhibited S100A11 membrane translocation, but little effect on S100A11 expression at total protein level. **f** Higher matrix stiffness strengthened S100A11 interaction with p67 phox and p47 phox. **g** ROS production in HCC cells transfected with pEGFP-OE-S100A11 or pFU-GW-shRNA-S100A11 in presence or absence of DPI under different stiffness stimulations. **h** Snail expression in HCC cells transfected with pEGFP-OE-S100A11 or pFU-GW-shRNA-S100A11 in presence or absence of DPI. Error bars indicate SD. *P < 0.05, **P < 0.01, ***P < 0.001

Obvious fibroblast-like morphology, greater migration ability, and F-actin polymerization under higher stiffness stimulation indicate that pseudopodial proteins may participate in stiffness-induced EMT in HCC cells. In Fig. 3a, overexpression of pseudopodial protein S100A11 or eIF4E upregulated N-cadherin, vimentin, and α-SMA expressions, but downregulated E-cadherin expression. On the contrary, suppression of them remarkably inhibited this mesenchymal shift (Fig. 3b). These results suggest that S100A11 and eIF4E may contribute to higher stiffness-induced EMT in HCC cells. Furthermore, knockdown of stiffness-sensor molecule integrin α5 or integrin β1 attenuated higher stiffness-induced mesenchymal shift, but S100A11 upregulation partially reversed this inhibitory effect and restored EMT phenotype (Additional file 4: Figure S3A, B), revealing a significant role of S100A11 in higher stiffness-induced EMT in HCC. Meanwhile, we found that distribution of S100A11 was from cytoplasm to membrane in HCC cells under higher stiffness stimulation (Fig. 3c, d), and knockdown of integrin α5 or integrin β1 attenuated the level of S100A11 in membrane protein (Fig. 3e). It indicates that there exists an obvious membrane translocation of S100A11 during higher stiffness-induced EMT in HCC.

NADPH oxidase (NOX) is an enzyme located at cell membrane and produces reactive oxygen species (ROS) which is associated with EMT [26, 28]. The expressions of NADPH oxidase subunits p47 phox and p67 phox in S100A11-overexpressed HCC cells were independent of substrate stiffness, but a Co-IP assay demonstrated that levels of p67 phox and p47 phox interacted with S100A11 were significantly upregulated in HCC cells under higher stiffness stimulation (Fig. 3f), suggesting that higher matrix stiffness strengthens S100A11 interaction with p67 phox and p47 phox and promotes NADPH oxidase assembly. Subsequently, we found an increase in ROS production in HCC cells under higher stiffness stimulation (Fig. 3g). Moreover, S100A11 upregulation promoted ROS production and Snail expression, but NOX inhibitor DPI suppressed this effect (Fig. 3g, h). Conversely, S100A11 downregulation inhibited ROS and Snail expression; however, DPI inhibitor could not strengthen this inhibition effect (Fig. 3g, h). All the data mentioned above illustrate that higher stiffness results in S100A11 trafficking to the plasma membrane, interacting with NADPH oxidase to increase ROS production and Snail expression, which contributes to EMT occurrence in HCC cells.

### eIF4E contributes to stiffness-induced EMT in HCC cells

**Fig. 4:**
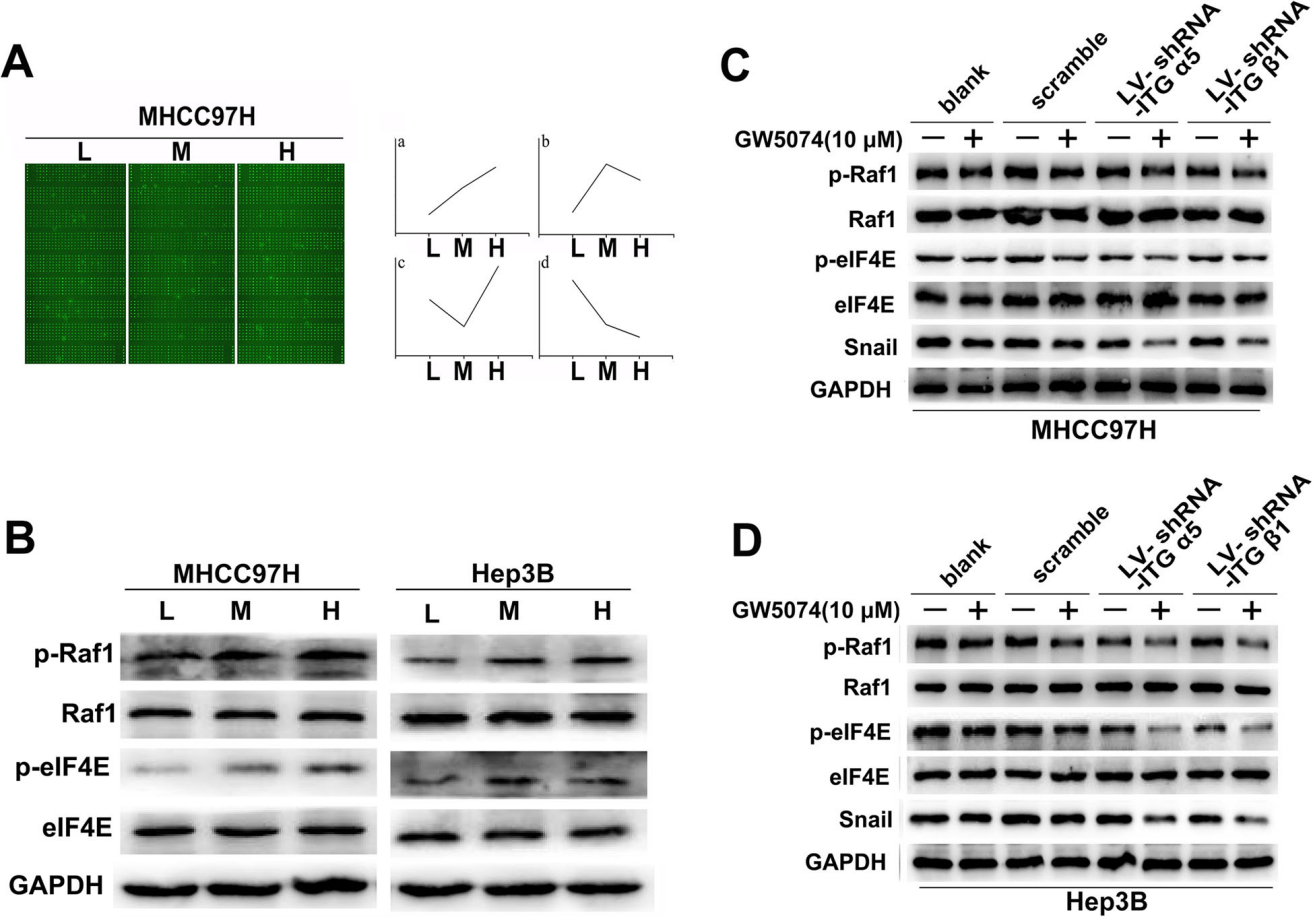
Integrin α5/β1-Raf1-eIF4E-Snail pathway contributes to higher stiffness-induced EMT in HCC cells. **a** Screening of differential phosphorylation levels of signal molecules in MHCC97H cells under different stiffness stimulation using cancer phosphorylation antibody arrays. L, low-stiffness substrate, 6 KPa; M, medium-stiffness substrate, 10 KPa; H, high-stiffness substrate, 16 KPa. The detailed expression patterns were listed in Additional file 8: Table S2–5. **b** Phosphorylation levels of Raf1 and eIF4E in HCC cells under different stiffness stimulations. **c** Inhibitor GW5074 suppressed the expressions of p-Raf1, p-eIF4E, and Snail in LV-shRNA-ITGβ1- or LV-shRNA-ITGα5-transfected MHCC97H cells grown on high stiffness substrate (16 KPa). **d** Inhibitor GW5074 suppressed the expressions of p-Raf1, p-eIF4E, and Snail in LV-shRNA-ITGβ1- or LV-shRNA-ITGα5-transfected Hep3B cells grown on high stiffness substrate (16 KPa). ITG, integrin

The eIF4E overexpression enhanced higher stiffness-induced EMT in HCC cells, while its knockdown attenuated the expression of EMT markers (Fig. 3a, b). Additionally, knockdown of integrin α5 or integrin β1 suppressed EMT, which could be partially rescued by elF4E upregulation (Additional file 5: S4A, S4B). Thus, elF4E may be involved in higher stiffness-induced EMT in HCC cells. We identified 131 differentially expressed phosphorylated proteins with different expression patterns related to higher stiffness stimulation in MHCC97H cells (Fig. 4a, Additional file 8: Table S2-S5), and enriched the upregulated molecules in 12 signaling pathways. Our previous work had validated the activation of Akt, Wnt, and JNK-c-Jun pathways and mTOR pathway in stiffness-mediated effects on gene expression and stemness [19–21, 23]. Here, higher stiffness upregulated the phosphorylation levels of Raf1 and elF4E in MHCC97H cells. Moreover, Raf1 was a leading differential phosphorylated protein in expression pattern 2 (Additional file 8: Table S3). Raf1-elF4E pathway has a regulation role in Snail expression [29]. Thus, we further detected the activation of Raf1-elF4E-snail pathway in stiffness-induced EMT and found that phosphorylation levels of Raf1 and elF4E, as well as Snail expression, were significantly increased in HCC cells under higher stiffness stimulation (Fig. 4b, Fig. 2g). Simultaneously, Raf1 inhibitor GW5074 obviously downregulated the phosphorylation of Raf1 and elF4E, as well as Snail expression (Fig. 4c, d), confirming a regulatory role of Raf1-elF4E-Snail pathway in stiffness-induced EMT. Knockdown of integrin α5 or β1 impaired the phosphorylation level of Raf1 and elF4E, as well as Snail expression (Fig. 4c, d), revealing that integrin α5 or β1 as an upstream molecule delivers stiffness signal into HCC cells. All these data suggest that Raf1 phosphorylation activates downstream molecules elF4E and Snail to regulate stiffness-induced EMT in HCC cells.

### Increased matrix stiffness enhances TGF β1 autocrine and triggers EMT occurrence in HCC cells

**Fig. 5:**
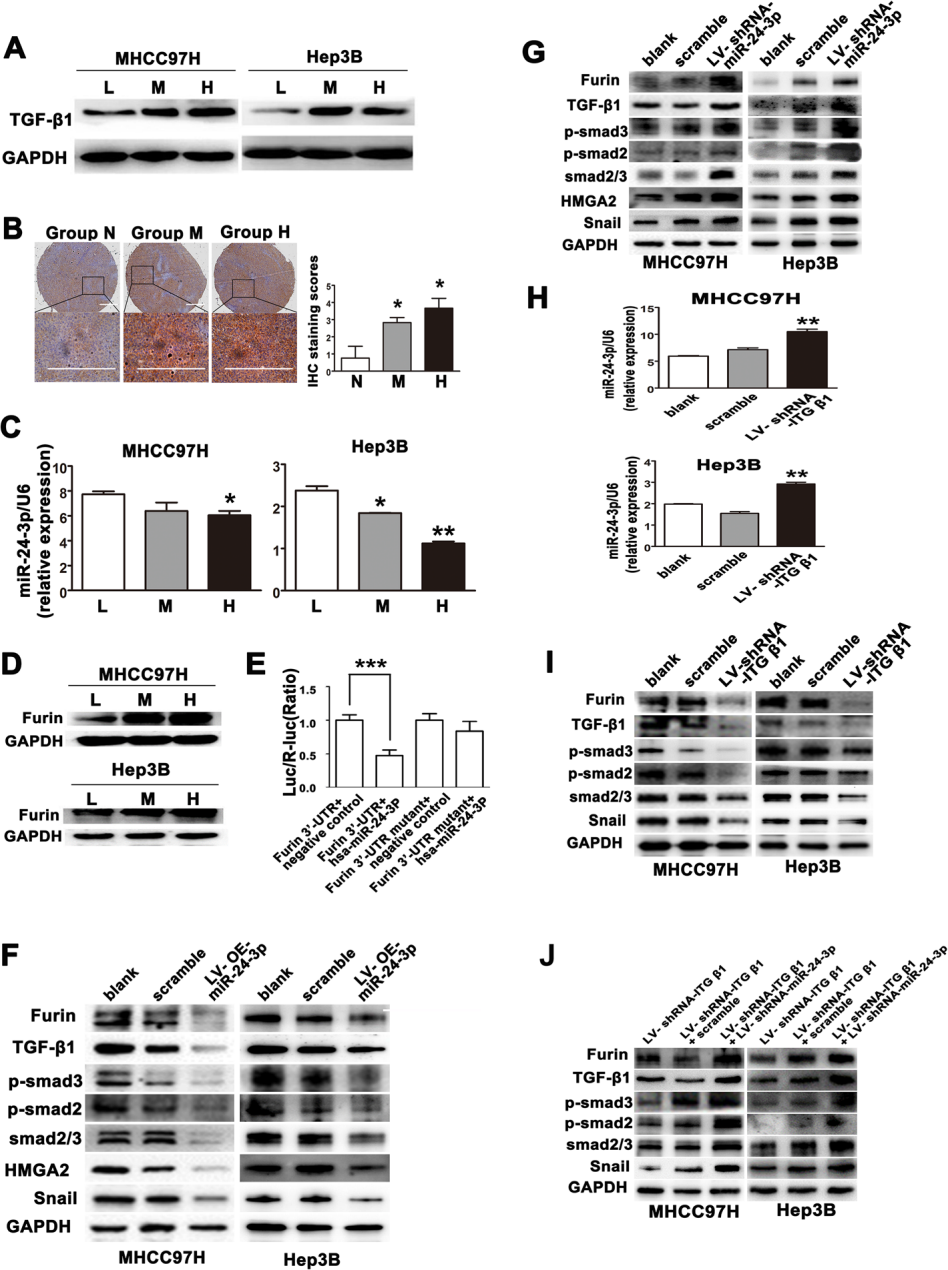
Increased matrix stiffness enhances TGF β1 autocrine and triggers EMT in HCC cells. **a** The expression of TGFβ1 in HCC cells under different stiffness stimulation. L, low-stiffness substrate, 6 KPa; M, medium-stiffness substrate, 10 KPa; H, high-stiffness substrate, 16 KPa. **b** TGFβ1 expression in orthotopic HCC tumors in group N, group M, and group H. N, normal stiffness; M, medium stiffness; H, high stiffness. Scale bar, 1000 μm. **c** miRNA-24-3p expression in HCC cells grown on different stiffness substrates. **d** Expression of Furin in HCC cells grown on different stiffness substrates. **e** Dual luciferase assay validated a specific regulation of miRNA-24-3p on Furin 3′UTR. **f** Expressions of Furin, TGFβ1, Smad2/3, p-Smad2, p-Smad3, HMGA2, and Snail in LV-miRNA-24-3p-overexpression-transfected HCC cells grown on high stiffness substrate (16 KPa). **g** Expressions of Furin, TGFβ1, Smad2/3, p-Smad2, p-Smad3, HMGA2, and Snail in LV-miRNA-24-3p-interference-transfected HCC cells grown on low stiffness substrate (6 KPa). **h** Suppression of integrin β1 resulted in an increase of miRNA-24-3p in HCC cells grown on high stiffness substrate (16 KPa). **i** Knockdown of integrin β1 suppressed the expressions of Furin, TGFβ1, Smad2/3, p-Smad2, and p-Smad3 in HCC cells grown on high stiffness substrate (16 KPa). **j** Inhibition of miRNA-24-3p reversed the expressions of Furin, TGFβ1, Smad2/3, p-Smad2, p-Smad3, HMGA2, and Snail in transfected HCC cells with LV-shRNA-ITGβ1 under higher stiffness stimulation. Error bars indicate SD. LV-OE-miR-24-3p, miRNA-24-3p-overexpression; LV-shRNA-miR-24-3p, miRNA-24-3p inhibition; *P < 0.05, **P < 0.01, ***P < 0.001

In spite of higher stiffness-triggered EMT in HCC cells independent of exogenous TGF β1 (Fig. 2e), we still could not exclude the existence of TGF β1 autocrine. Our results showed an obvious increase in TGF β1 expression in HCC cells cultured on higher stiffness substrate (Fig. 5a) and in tumor tissues from HCC models with higher liver stiffness background (Fig. 5b). Simultaneously, higher stiffness stimulation also elevated the level of TGFβ1 in culture medium supernatant of HCC cells (Additional file 6: Figure S5), indicating that higher matrix stiffness may promote TGF β1 autocrine. Considering that extracellular mechanical signals can influence miRNA expression [30, 31], we investigated whether a specific miRNA was involved in stiffness-induced TGF β1 autocrine. The miRNA-24-3p predicted by mirTarBase database might indirectly influence TGF β1 level. As shown in Fig. 5c, d, higher stiffness significantly downregulated the expression of miRNA-24-3p and upregulated the expression of its target protein Furin. Dual luciferase assay revealed a specific regulation of miRNA-24-3p on Furin 3′UTR (Fig. 5e). Furin is responsible for pro-TGF-β1 proteolytic processing. Thus, miRNA-24-3p and its target protein Furin might participate in higher stiffness-enhanced TGF β1 autocrine. Under higher stiffness stimulation, miRNA-24-3p-overexpressed HCC cells exhibited distinct decreases in the expressions of Furin, TGF β1, Smad2/3, p-Smad2, p-Smad3, Snail, and HMGA2 (Fig. 5f). Inversely, under lower stiffness stimulation, miRNA-24-3p-shRNA HCC cells presented significant increases in the expressions of the molecules mentioned above (Fig. 5g). Additionally, suppression of integrin β1 resulted in an increase of miRNA-24-3p (Fig. 5h) and a decrease of Furin, TGF β1, Smad2/3 phosphorylation, and Snail in HCC cells grown on higher stiffness substrate (Fig. 5i), but inhibition of miRNA-24-3p reversed the above corresponding changes (Fig. 5j). All the results suggest that miRNA-24-3p participates in stiffness-mediated TGF β1 autocrine and is responsible for higher expression of Snail and EMT occurrence in HCC cells.

### Clinical significance of liver stiffness and the molecules responsible for stiffness-induced EMT in HCC cohort

**Fig. 6:**
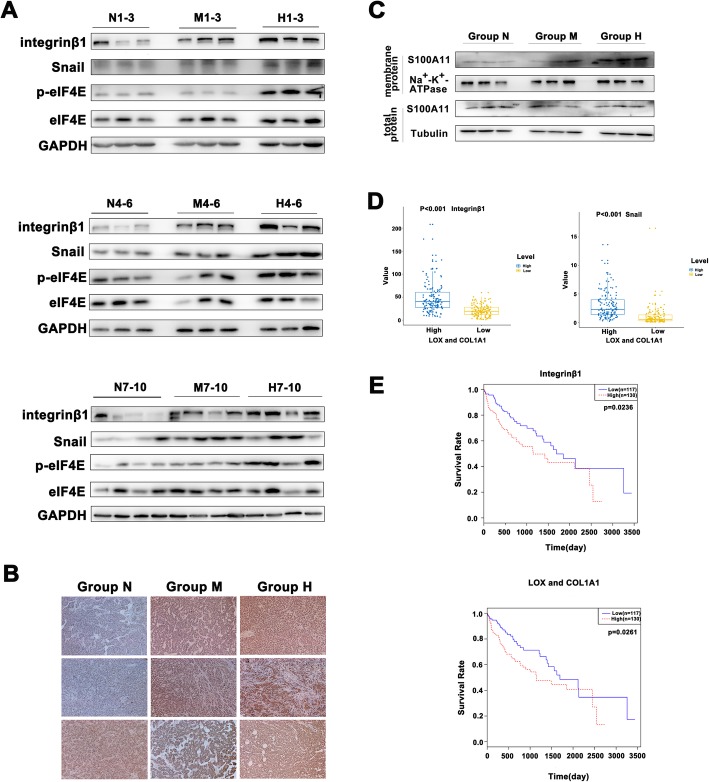
Clinical significance of liver stiffness and the molecules responsible for stiffness-induced EMT in HCC cohorts. **a** Integrin β1, Snail, and eIF4E phosphorylation were significantly overexpressed in clinical HCC tissues from higher liver stiffness group compared with those from normal liver stiffness group. N, normal liver stiffness; M, medium liver stiffness; H, high liver stiffness. **b** The levels of TGF β1 expression were markedly increased in higher liver stiffness group compared with that in normal liver stiffness group. **c** The levels of S100A11 in membrane subfractions were significantly increased in higher liver stiffness groups. **d** Integrin β1 or Snail was significantly overexpressed in the high-stiffness group (LOX^high^/Collagen I^high^, *n* = 130) compared with the low-stiffness group (LOX^low^/Collagen I^low^, *n* = 117) in TCGA-HCC cohort. **e** Analysis of the survival curve showed that high expression of LOX/Collage or integrin β1 was significantly associated with poor overall survival in TCGA-HCC patients

To validate the clinical implication of liver stiffness, we analyzed clinicopathologic data of 74 HCC patients in our hospital and found that higher liver stiffness was not only correlated with gender, albumin, conjugated bilirubin, AST, GGT, and Metavir’s G grade and S grade significantly, but more importantly closely associated with poor tumor differentiation (*P* = 0.026) and higher HCC recurrence (*P* = 0.036) (Table 1), demonstrating that increased liver stiffness promotes a loss of tumor differentiation and higher recurrence. Additionally, the proteins required for stiffness-induced EMT such as integrin β1, Snail, and eIF4E phosphorylation showed a significant upregulation in HCC tissues from higher liver stiffness group (Fig. 6a). Meanwhile, TGF β1 secretion and S100A11 level at membrane subfractions also had an obvious increase trend (Fig. 6b, c), in agreement with the findings from cell experiment and animal models. Next, we further downloaded the clinical data of 424 HCC samples and their gene expression data (integrin β1, LOX, Collagen I, Snail) from The Cancer Genome Atlas (TCGA). Taking the median expression level of LOX and Collagen I as a threshold, we classified the samples into two groups such as the low-stiffness group (LOX^low^/Collagen I^low^, 117 patients) and the high-stiffness group (LOX^high^/Collagen I^high^, 130 patients). Compared with that in the low-stiffness group, integrin β1 or Snail is highly expressed in the high-stiffness group (*P* < 0.001, Fig. 6d), indicating increased matrix stiffness is positively correlated with the expressions of integrinβ1 and Snail. Subsequently we clarified a significance between the gene expression level (integrinβ1 or LOX/Collagen I) and overall survival time in the HCC cohort (*P* < 0.05, Fig. 6e), revealing that high expression of stiffness-related marker (integrin β1 or LOX/Collage I) better indicates unfavorable survival of HCC patients. All the above data confirm the clinical significance of high liver stiffness and the molecules for higher stiffness-induced EMT (integrin β1, Snail, eIF4E, TGFβ1, and S100A11) in HCC progression and unfavorable outcome.

**Table 1:** Associations of liver stiffness with clinicopathologic characteristics in HCC patients

	Liver stiffness (kPa)			*P* value
	≤ 8	> 8, < 12	≥ 12	
No. of patients	18	21	35	
Age (years)	52.56 ± 13.84	55.81 ± 10.68	55.09 ± .10.02	0.639
Gender (male/female)	10/8	18/3	31/4	0.013
AFP (ng/ml)	5846 ± 14,403	4683 ± 13,468	7123 ± 17,313	0.412
ALB (g/l)	43.01 ± 3.21	44.78 ± 3.53	41.37 ± 4.08	0.006
CB (umol/l)	4.02 ± 1.60	4.77 ± 2.22	6.44 ± 5.97	0.032
ALT (U/l)	31.18 ± 24.15	34.47 ± 22.15	42.58 ± 48.63	0.245
AST (U/l)	27.43 ± 18.81	30.59 ± 17.68	39.11 ± 34.43	0.035
GGT (U/l)	59.42 ± 54.44	79.63 ± 63.29	128.34 ± 180.10	0.020
ALP (U/l)	84.88 ± 35.79	87.14 ± 28.71	101.26 ± 75.53	0.810
APTT (second)	22.54 ± 11.93	20.60 ± 8.93	24.84 ± 8.66	0.162
Tumor size (cm)	5.68 ± 2.34	6.35 ± 4.45	7.41 ± 5.06	0.616
Tumor encapsulation				0.483
Yes	12	10	19	
No	6	11	16	
Microvascular invasion				0.615
Yes	12	11	19	
No	6	10	16	
Tumor differentiation				0.026
Edmondson I–II	13	18	18	
III–IV	5	3	17	
Metavir’s G grade				0.040
0–1	8	8	8	
2–3	10	13	27	
Metavir’s S grade				< 0.001
0–2	12	12	3	
3–4	6	9	32	
Recurrence				0.036
Yes	3	4	16	
No	15	17	19	

*SD* standard deviation, *AFP* A-fetoprotein, *ALB* albumin, *CB* conjugated bilirubin, *ALT* alanine aminotransferase, *AST* aspartate aminotransferase, *GGT* γ-glutamyltransferase, *ALP* alkaline phosphatase, *APTT* activated partial thromboplastin time

## Discussion

Liver stiffening as a remarkable pathological phenotype frequently emerges in the late stage of chronical liver diseases and HCC. Now, increased liver stiffness has become an important clinicopathological parameter of HCC to indicate its pathological grade and the risk of unfavorable outcome [27, 32]. Although previous studies have suggested a dominant regulatory role of matrix stiffness in invasion/metastasis gene expression, stemness, and pre-metastatic niche formation of HCC [19–21, 23], the underlying mechanisms of matrix stiffness regulating HCC invasion and metastasis remain largely unexplored. Proteins required for pseudopod formation and cytoskeleton remodeling have long been associated with malignancy [33, 34], and individual pseudopod proteins can alter the morphology of cancer cells and influence the E-cadherin expression and nuclear translocation of Smads and Snail [35]. Given that morphology and malignant properties of HCC cells grown on higher stiffness substrate were very similar to those of tumor cells undergoing EMT, we further explored whether matrix stiffness alone was sufficient to drive EMT in HCC cells and whether pseudopod-associated proteins were involved in this pathological process.

We first established buffalo rat HCC models with different liver stiffness backgrounds to clarify the relationship between liver stiffness and HCC progression. The data suggested that higher liver stiffness facilitated HCC growth and enhanced its metastasis. Subcutaneous tumor analysis also showed an obvious acceleration in HCC growth and invasion/metastasis gene expression in a group of higher matrix stiffness. In vitro*,* HCC cells exhibited a fibroblast-like morphology, greater motility ability, and higher expression of metastasis-associated genes under higher stiffness stimulation. These alterations clearly stated that matrix stiffness signals strengthen the invasion and metastasis of HCC and modulate its malignant properties. More importantly, in the absence of exogenous TGF β1, HCC cells also acquired an obvious mesenchymal attribute grown on higher stiffness substrate. Meanwhile, the suppression of integrin β1 or α5 reversed the expressions of EMT markers. All these findings strongly support that matrix stiffness alone is sufficient to drive EMT in HCC cells, in agreement with results in breast cancer cells [36]. As expected, the inhibition of S100A11 and eIF4E strikingly attenuated higher stiffness-induced EMT whereas the overexpression of them facilitated this transition, indicating that pseudopod-associated protein S100A11 and eIF4E may participate in stiffness-induced EMT. S100A11 is highly expressed in various cancers [37–40] and related to poor differentiation, distant metastasis, and shorter disease-free survival [40]. S100A11 promotes the invasion and migration of HCC cells [38] and is required for TGF-initiated EMT in colorectal cancer cells [39]. Here, higher stiffness stimulation induced an obvious membrane translocation of S100A11; this change resulted in an interaction between S100A11 and NADPH oxidase which promoted ROS production. ROS increases the hypermethylation of E-cadherin promoter [41], while ROS suppression reverses eIF5A2-induced EMT in HCC cells [42]. Our results validated that S100A11 downregulation suppressed ROS production and Snail expression, suggesting a positive correlation between ROS and EMT. Taken together, higher matrix stiffness alone initiates EMT in HCC cells via integrin-mediated S100A11 localization.

In addition, we found that another pseudopod protein eIF4E was also involved in stiffness-induced EMT in HCC. eIF4E highly expresses in a variety of human malignancies and is relevant to cancer development and progression [43, 44]. elF4E antagonist inhibits TGFβ1-initiated EMT in lung epithelial cells [45]. The elF4E phosphorylation regulates the expression of Snail and MMP-3 and in turn controls EMT [46]. The results in our study revealed that phosphorylation levels of elF4E and its upstream molecule Raf1 were all significantly upregulated in HCC cells under higher stiffness stimulation, which could be attenuated by Raf1 inhibitor GW5074. Meanwhile, the inhibition of integrin α5 or β1 downregulated the phosphorylation levels of Raf1 and elF4E, as well as Snail expression. Accordingly, integrinα5/β1-Raf1-elF4E-Snail pathway participates in higher stiffness-induced EMT in HCC cells.

Considering that there was a significant increase in Smad2/3 phosphorylation level under higher stiffness stimulation, we continued to analyze whether TGFβ1 autocrine existed in HCC cells during this pathological process. A loss or gain of miRNAs always occurs in EMT-increased tumor invasion and metastasis [47]. Matrix rigidity increase potentiates TGFβ-induced miR-181a expression and enhances metastasis of malignant mammary epithelial cells [48]. A mechanically regulated miR-18a targets tumor suppressor PTEN and promotes breast malignancy [31]. Here, we first confirmed the existence of TGFβ1 autocrine in HCC cells under higher stiffness stimulation at cell and tissue levels, and then predicted that miRNA-24-3p might indirectly influence TGFβ1 level. Under higher stiffness stimulation, HCC cells presented a significant loss of miRNA-24-3p, but having an increase in target gene Furin. Furthermore, inhibition of miRNA-24-3p promoted the expressions of Furin, TGFβ1, Smad2/3 phosphorylation, Snail, and HMGA2, whereas miRNA-24-3p overexpression showed an opposite trend. Additionally, the knockdown of integrin β1 suppressed the expressions of Furin, TGF β1, Smad2/3 phosphorylation, and Snail in HCC cells grown on higher stiffness, but the inhibition of miRNA-24-3p reversed the above changes. Altogether, integrin β1/miRNA-24-3p/Furin/TGF β1 pathway also contributes to stiffness-induced EMT.

Subsequently, a clinical significance of higher liver stiffness in pathological grade and HCC prognosis was validated in HCC samples and TCGA analysis, and the potential values of the required proteins for higher stiffness-induced EMT were clarified in indicating poor tumor differentiation and HCC recurrence.

Although the three signaling pathways described above participate in higher stiffness-induced EMT in HCC metastasis, we are still unable to exclude the existence of other signal pathways in this pathological process. With the development of high-throughput signal pathway screening technology and the establishment of ideal animal model, other molecular mechanisms related to stiffness-induced EMT will be better elucidated.

**Fig. 7:**
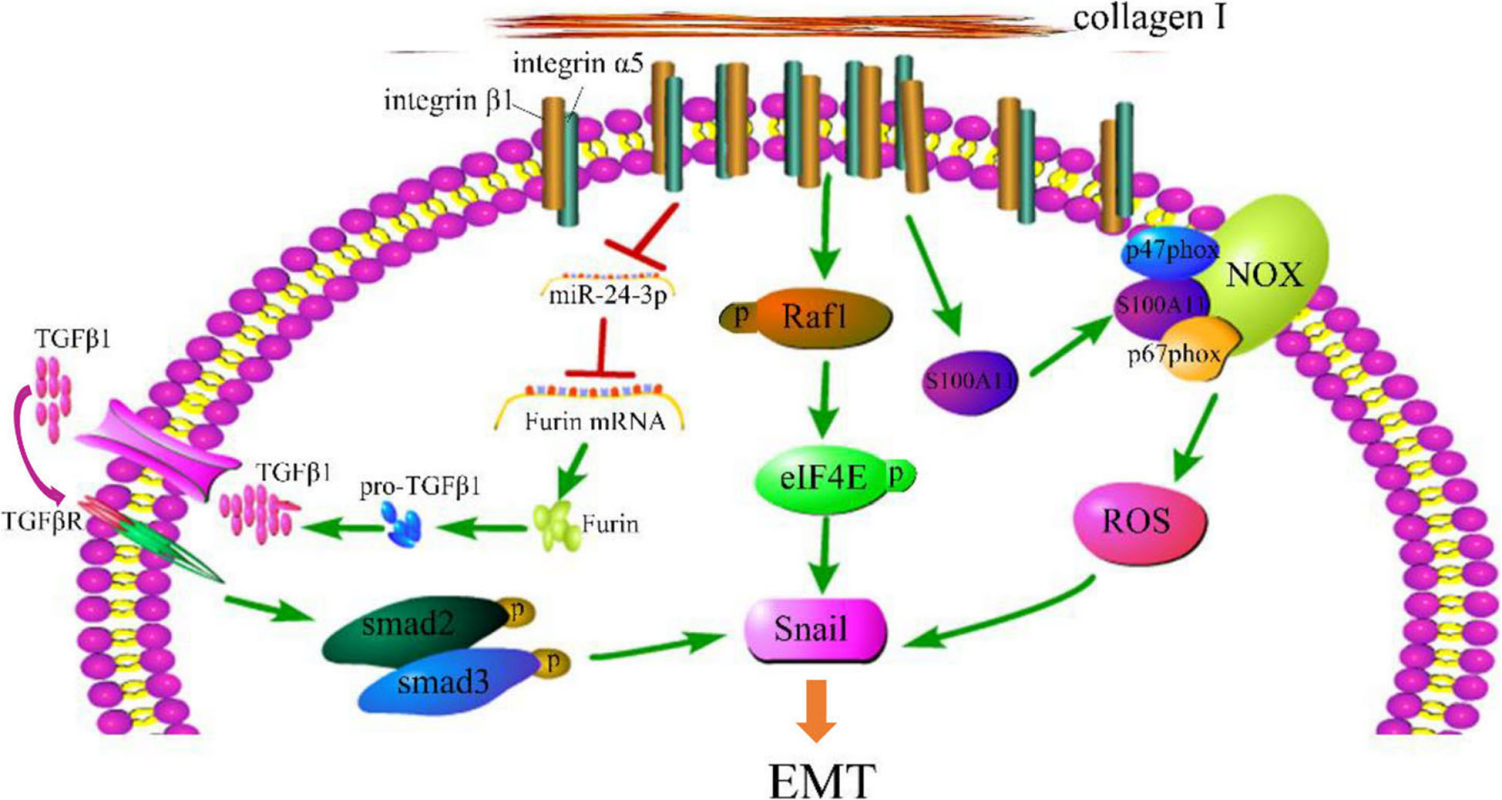
Schematic diagram of the proposed mechanism by which higher matrix stiffness drives EMT in HCC cells independently

In summary, the three signaling pathways converging on Snail expression contribute to higher stiffness-induced EMT in HCC metastasis, including S100A11 membrane translocation, eIF4E phosphorylation, and TGFβ1 autocrine (Fig. 7). To the best of our knowledge, this is the first report to elucidate the molecular mechanisms by which matrix stiffness as an independent initiator triggers EMT occurrence in HCC cells. This finding may implicate the clinical use of liver stiffness as an intervention target to thwart HCC metastasis.

## Supplementary information

**Additional file 1:.** Supporting Information.

**Additional file 2: Figure S1.** Higher liver stiffness promotes the growth of HCC and facilitates HCC invasion and metastasis in vivo. (A) The mRNA expression of integrin β1, LOX and AFP in orthotopic HCC tumors in groups N, M and H. (B) Liver function analysis of buffalo rat HCC models with different liver stiffness backgrounds. B, Healthy buffalo rats, C, HCC buffalo rats with normal liver stiffness, M, HCC buffalo rats with medium liver stiffness, S, HCC buffalo rats with high liver stiffness. (C) Gross appearance and wet weight of subcutaneous tumors derived from Hep3B cells mixed in Matrigel and varied concentration of COL1. (D) Histochemistry analysis of subcutaneous tumors and their Ki-67 expression. (E) The expressions of integrin β1 and FAK in subcutaneous tumors. (F) The expressions of MMP2, MMP9, SPP1, CD44 in subcutaneous tumors. C, Hep3B cells; Matr., Hep3B cells in Matrigel; Matr+L., Hep3B cells in Matrigel and low concentration COL1; Matr+H., Hep3B cells in Matrigel and high concentration COL1.

**Additional file 3: Figure S2.** An in vitro system of COL1-coated polyacrylamide gels with tunable stiffness. (A) Preparation of an in vitro system of COL1-coated polyacrylamide gels with tunable stiffness (B) Stiffness values of different stiffness substrates (C) Morphology alteration of HCC cells grown on FN/LN- coated gels with tunable stiffness and their Snail expression. (D) Expressions of integrin α5 or integrin β1 in HCC cells transfected with LV-shRNA-ITG α5 and LV-shRNA-ITG β1.

**Additional file 4: Figure S3.** S100A11 participates in stiffness-induced EMT in HCC cells. (A) The expression of EMT markers in HCC cells co-transfected with pFU-GW-shRNA-S100A11 and LV-shRNA-ITGβ1 or pEGFP-OE-S100A11 and LV-shRNA-ITGβ1 under higher stiffness stimulation. (B) The expression of EMT markers in HCC cells co-transfected with pFU-GW-shRNA-S100A11 and LV-shRNA-ITGα5 or pEGFP-OE-S100A11 and LV-shRNA-ITGα5 under higher stiffness stimulation.

**Additional file 5: Figure S4.** eIF4E participates in stiffness-induced EMT in HCC cells. (A) The expression of EMT markers in HCC cells co-transfected with pFU-GW-shRNA- eIF4E and LV-shRNA-ITGα5 or pEGFP-OE-eIF4E overexpression and LV-shRNA-ITGα5 under higher stiffness stimulation (B) The expression of EMT markers in HCC cells co-transfected with pFU-GW-shRNA- eIF4E and LV-shRNA-ITGα5 or pEGFP-OE-eIF4E and LV-shRNA-ITGα5 under higher stiffness stimulation.

**Additional file 6: Figure S5.** The levels of TGF-β1 in culture supernatants of HCC cells grown on different stiffness substrates.

**Additional file 7: Table S1.** Primer pairs used for qRT-PCR

**Additional file 8: Table S2**. Different phosphorylation signal proteins in expression pattern of “a”. **Table S3**. Different phosphorylation signal proteins in expression pattern of“b”. **Table S4**. Different phosphorylation signal proteins in expression pattern of “c”. **Table S5**. Different phosphorylation signal proteins in expression pattern of “d”.

## Data Availability

The data in the current study are available from the corresponding author on reasonable request.

## Abbreviations

CCl4: Carbon tetrachloride
COLI: Collagen I
eIF4E: Eukaryotic initiation factor 4E
EMT: Epithelial-mesenchymal transition
HCC: Hepatocellular carcinoma
LOX: Lysyl oxidase
MMPs: Matrix metalloproteinases
OPN: Osteopontin
TGF β1: Transforming growth factor β1
VEGF: Vascular endothelial growth factor

## References

[1] Yang JD, Nakamura I, Roberts LR (2011). The tumor microenvironment in hepatocellular carcinoma: current status and therapeutic targets. Semin Cancer Biol.

[2] Oskarsson T (2013). Extracellular matrix components in breast cancer progression and metastasis. Breast.

[3] Cretu A, Brooks PC (2007). Impact of the non-cellular tumor microenvironment on metastasis: potential therapeutic and imaging opportunities. J Cell Physiol.

[4] Levental KR, Yu H, Kass L, Lakins JN, Egeblad M, Erler JT, Fong SF, Csiszar K, Giaccia A, Weninger W, Yamauchi M, Gasser DL, Weaver VM (2009). Matrix crosslinking forces tumor progression by enhancing integrin signaling. Cell..

[5] Leight JL, Wozniak MA, Chen S, Lynch ML, Chen CS (2012). Matrix rigidity regulates a switch between TGF-β1-induced apoptosis and epithelial-mesenchymal transition. Mol Biol Cell.

[6] Pelham RJ, Wang Y (1997). Cell locomotion and focal adhesions are regulated by substrate flexibility. Proc Natl Acad Sci U S A.

[7] Engler AJ, Sen S, Sweeney HL, Discher DE (2006). Matrix elasticity directs stem cell lineage specification. Cell..

[8] Klein EA, Yin L, Kothapalli D, Castagnino P, Byfield FJ, Xu T, Levental I, Hawthorne E, Janmey PA, Assoian RK (2009). Cell-cycle control by physiological matrix elasticity and in vivo tissue stiffening. Curr Biol.

[9] Tilghman RW, Cowan CR, Mih JD, Koryakina Y, Gioeli D, Slack-Davis JK, Blackman BR, Tschumperlin DJ, Parsons JT (2010). Matrix rigidity regulates cancer cell growth and cellular phenotype. PLoS One.

[10] Tilghman RW, Blais EM, Cowan CR, Sherman NE, Grigera PR, Jeffery ED, Fox JW, Blackman BR, Tschumperlin DJ, Papin JA, Parsons JT (2012). Matrix rigidity regulates cancer cell growth by modulating cellular metabolism and protein synthesis. PLoS One.

[11] Kshitiz PJ, Kim P, Helen W, Engler AJ, Levchenko A, Kim DH (2012). Control of stem cell fate and function by engineering physical microenvironments. Integr Biol (Camb).

[12] Keely PJ (2011). Mechanisms by which the extracellular matrix and integrin signaling act to regulate the switch between tumor suppression and tumor promotion. J Mammary Gland Biol Neoplasia.

[13] Yeung T, Georges PC, Flanagan LA, Marg B, Ortiz M, Funaki M, Zahir N, Ming W, Weaver V, Janmey PA (2005). Effects of substrate stiffness on cell morphology, cytoskeletal structure, and adhesion. Cell Motil Cytoskeleton.

[14] Fattovich G, Stroffolini T, Zagni I, Donato F (2004). Hepatocellular carcinoma in cirrhosis: incidence and risk factors. Gastroenterology..

[15] Greten TF, Papendorf F, Bleck JS, Kirchhoff T, Wohlberedt T, Kubicka S, Klempnauer J, Galanski M, Manns MP (2005). Survival rate in patients with hepatocellular carcinoma: a retrospective analysis of 389 patients. Br J Cancer.

[16] Jung KS, Kim SU, Choi GH, Park JY, Park YN, do Kim Y, Ahn SH, Chon CY, Kim KS, Choi EH, Choi JS, Han KH (2012). Prediction of recurrence after curative resection of hepatocellular carcinoma using liver stiffness measurement (FibroScan®). Ann Surg Oncol.

[17] Cescon M, Colecchia A, Cucchetti A, Peri E, Montrone L, Ercolani G, Festi D, Pinna AD (2012). Value of transient elastography measured with FibroScan in predicting the outcome of hepatic resection for hepatocellular carcinoma. Ann Surg.

[18] Schrader J, Gordon-Walker TT, Aucott RL, van Deemter M, Quaas A, Walsh S, Benten D, Forbes SJ, Wells RG, Iredale JP (2011). Matrix stiffness modulates proliferation, chemotherapeutic response, and dormancy in hepatocellular carcinoma cells. Hepatology..

[19] Dong Y, Xie X, Wang Z, Hu C, Zheng Q, Wang Y, Chen R, Xue T, Chen J, Gao D, Wu W, Ren Z, Cui J (2014). Increasing matrix stiffness upregulates vascular endothelial growth factor expression in hepatocellular carcinoma cells mediated by integrin β1. Biochem Biophys Res Commun.

[20] You Y, Zheng Q, Dong Y, Wang Y, Zhang L, Xue T, Xie X, Hu C, Wang Z, Chen R, Wang Y, Cui J, Ren Z (2015). Higher matrix stiffness upregulates osteopontin expression in hepatocellular carcinoma cells mediated by integrin β1/GSK3β/β-catenin signaling pathway. PLoS One.

[21] You Y, Zheng Q, Dong Y, Xie X, Wang Y, Wu S, Zhang L, Wang Y, Xue T, Wang Z, Chen R, Wang Y, Cui J, Ren Z (2016). Matrix stiffness-mediated effects on stemness characteristics occurring in HCC cells. Oncotarget..

[22] Zhao G, Cui J, Qin Q, Zhang J, Liu L, Deng S, Wu C, Yang M, Li S, Wang C (2010). Mechanical stiffness of liver tissues in relation to integrin β1 expression may influence the development of hepatic cirrhosis and hepatocellular carcinoma. J Surg Oncol.

[23] Wu S, Zheng Q, Xing X, Dong Y, Wang Y, You Y, Chen R, Hu C, Chen J, Gao D, Zhao Y, Wang Z, Xue T, Ren Z, Cui J (2018). Matrix stiffness-upregulated LOXL2 promotes fibronectin production, MMP9 and CXCL12 expression and BMDCs recruitment to assist pre-metastatic niche formation. J Exp Clin Cancer Res.

[24] O'Connor JW, Gomez EW (2014). Biomechanics of TGFβ-induced epithelial-mesenchymal transition: implications for fibrosis and cancer. Clin Transl Med.

[25] Thiery JP, Acloque H, Huang RY, Nieto MA (2009). Epithelial-mesenchymal transitions in development and disease. Cell..

[26] Lee K, Chen QK, Lui C, Cichon MA, Radisky DC, Nelson CM (2012). Matrix compliance regulates Rac1b localization, NADPH oxidase assembly, and epithelial-mesenchymal transition. Mol Biol Cell.

[27] Wong VW, Vergniol J, Wong GL, Foucher J, Chan HL, Le Bail B, Choi PC, Kowo M, Chan AW, Merrouche W, Sung JJ, de Lédinghen V (2010). Diagnosis of fibrosis and cirrhosis using liver stiffness measurement in nonalcoholic fatty liver disease. Hepatology..

[28] Karicheva O, Rodriguez-Vargas JM, Wadier N, Martin-Hernandez K, Vauchelles R, Magroun N, Tissier A, Schreiber V, Dantzer F (2016). PARP3 controls TGFβ and ROS driven epithelial-to-mesenchymal transition and stemness by stimulating a TG2-Snail-E-cadherin axis. Oncotarget..

[29] Romagnoli M, Belguise K, Yu Z, Wang X, Landesman-Bollag E, Seldin DC, Chalbos D, Barillé-Nion S, Jézéquel P, Seldin ML, Sonenshein GE (2012). Epithelial-to-mesenchymal transition induced by TGF-β1 is mediated by Blimp-1-dependent repression of BMP-5. Cancer Res.

[30] Le LT, Cazares O, Mouw JK, Chatterjee S, Macias H, Moran A, Ramos J, Keely PJ, Weaver VM, Hinck L (2016). Loss of miR-203 regulates cell shape and matrix adhesion through ROBO1/Rac/FAK in response to stiffness. J Cell Biol.

[31] Mouw JK, Yui Y, Damiano L, Bainer RO, Lakins JN, Acerbi I, Ou G, Wijekoon AC, Levental KR, Gilbert PM, Hwang ES, Chen YY, Weaver VM (2014). Tissue mechanics modulate microRNA-dependent PTEN expression to regulate malignant progression. Nat Med.

[32] Park MS, Han KH, Kim SU (2014). Non-invasive prediction of development of hepatocellular carcinoma using transient elastography in patients with chronic liver disease. Expert Rev Gastroenterol Hepatol.

[33] Yu X, Zech T, McDonald L, Gonzalez EG, Li A, Macpherson I, Schwarz JP, Spence H, Futó K, Timpson P, Nixon C, Ma Y, Anton IM, Visegrády B, Insall RH, Oien K, Blyth K, Norman JC, Machesky LM (2012). N-WASP coordinates the delivery and F-actin-mediated capture of MT1-MMP at invasive pseudopods. J Cell Biol.

[34] García E, Ragazzini C, Yu X, Cuesta-García E, Bernardino de la Serna J, Zech T, Sarrió D, Machesky LM, Antón IM (2016). WIP and WICH/WIRE co-ordinately control invadopodium formation and maturation in human breast cancer cell invasion. Sci Rep.

[35] Shankar J, Messenberg A, Chan J, Underhill TM, Foster LJ, Nabi IR (2010). Pseudopodial actin dynamics control epithelial-mesenchymal transition in metastatic cancer cells. Cancer Res.

[36] Wei SC, Fattet L, Tsai JH, Guo Y, Pai VH, Majeski HE, Chen AC, Sah RL, Taylor SS, Engler AJ, Yang J (2015). Matrix stiffness drives epithelial -mesenchymal transition and tumour metastasis through a TWIST1-G3BP2 mechanotransduction pathway. Nat Cell Biol.

[37] Ohuchida K, Mizumoto K, Ohhashi S, Yamaguchi H, Konomi H, Nagai E, Yamaguchi K, Tsuneyoshi M, Tanaka M (2006). S100A11, a putative tumor suppressor gene, is overexpressed in pancreatic carcinogenesis. Clin Cancer Res.

[38] Luo X, Xie H, Long X, Zhou M, Xu Z, Shi B, Jiang H, Li Z (2013). EGFRvIII mediates hepatocellular carcinoma cell invasion by promoting S100 calcium binding protein A11 expression. PLoS One.

[39] Niu Y, Shao Z, Wang H, Yang J, Zhang F, Luo Y, Xu L, Ding Y, Zhao L (2016). LASP1-S100A11 axis promotes colorectal cancer aggressiveness by modulating TGFβ/Smad signaling. Sci Rep.

[40] Woo T, Okudela K, Mitsui H, Tajiri M, Rino Y, Ohashi K, Masuda M (2015). Up-regulation of S100A11 in lung adenocarcinoma - its potential relationship with cancer progression. PLoS One.

[41] Lim SO, Gu JM, Kim MS, Kim HS, Park YN, Park CK, Cho JW, Park YM, Jung G (2008). Epigenetic changes induced by reactive oxygen species in hepatocellular carcinoma: methylation of the E-cadherin promoter. Gastroenterology..

[42] Liu RR, Lv YS, Tang YX, Wang YF, Chen XL, Zheng XX, Xie SZ, Cai Y, Yu J, Zhang XN (2016). Eukaryotic translation initiation factor 5A2 regulates the migration and invasion of hepatocellular carcinoma cells via pathways involving reactive oxygen species. Oncotarget..

[43] Jiang XM, Yu XN, Huang RZ, Zhu HR, Chen XP, Xiong J, Chen ZY, Huang XX, Shen XZ, Zhu JM (2016). Prognostic significance of eukaryotic initiation factor 4E in hepatocellular carcinoma. J Cancer Res Clin Oncol.

[44] Khosravi S, Tam KJ, Ardekani GS, Martinka M, McElwee KJ, Ong CJ (2015). eIF4E is an adverse prognostic marker of melanoma patient survival by increasing melanoma cell invasion. J Invest Dermatol.

[45] Smith KA, Zhou B, Avdulov S, Benyumov A, Peterson M, Liu Y, Okon A, Hergert P, Braziunas J, Wagner CR, Borok Z, Bitterman PB (2015). Transforming growth factor-β1 induced epithelial mesenchymal transition is blocked by a chemical antagonist of translation factor eIF4E. Sci Rep.

[46] Robichaud N, del Rincon SV, Huor B, Alain T, Petruccelli LA, Hearnden J, Goncalves C, Grotegut S, Spruck CH, Furic L, Larsson O, Muller WJ, Miller WH, Sonenberg N (2015). Phosphorylation of eIF4E promotes EMT and metastasis via translational control of SNAIL and MMP-3. Oncogene..

[47] Tang J, Li Y, Wang J, Wen Z, Lai M, Zhang H (2016). Molecular mechanisms of microRNAs in regulating epithelial-mesenchymal transitions in human cancers. Cancer Lett.

[48] Taylor MA, Sossey-Alaoui K, Thompson CL, Danielpour D, Schiemann WP (2013). TGF-β upregulates miR-181a expression to promote breast cancer metastasis. J Clin Invest.

