# Supplementary material for: Higher matrix stiffness as an independent initiator triggers epithelial-mesenchymal transition and facilitates HCC metastasis
Source: J Hematol Oncol. 2019 Nov 8;12:112. doi: 10.1186/s13045-019-0795-5 (PMC6839087; doi:10.1186/s13045-019-0795-5)
Supplement: Supplementary file 1 — Additional file 1:. Supporting Information. [file 13045_2019_795_MOESM1_ESM.docx]

**Supporting information**

**HCC cells and cell culture**

Highly-metastatic MHCC97H and low-metastatic Hep3B cells were grown in Dulbecco’s Modiﬁed Eagle’s Medium (Gibco, Gaithersburg, USA) supplemented with 10% fetal bovine serum (FBS, Biowest, South America Origin) and in minimum essential medium (Gibco, Gaithersburg, USA) supplemented with 10% FBS, respectively. McA-RH7777 cells, obtained from the American Type Culture Collection, were grown in the same culture medium as MHCC97H cells.

**Preparation of in vitro system of mechanically tunable COL1-coated polyacrylamide gel**

In brief, 10% acrylamide (Acr), 0.01% to 0.5% bis-acrylamide (Bis) in a HEPES-buffered solution (pH 8), 10% ammonium persulfate (APS, 1/100 volume) and TEMED (1/100 volume) were mixed and polymerized to make 1 mm-thick uniform flat PAGE gels with different stiffness levels. The rigidity of the PAGE gels were measured using a TA.Xtplus Texture Analyzer, and the stiffness values of different gels as shown in Fig 2A. Then, a gel was cut into quadrate gels with a size of 4 cm×5 cm suitable for a culture dish. About 320µl COL1 solution (0.1 mg/ml) was spread onto the gel for 90 min crosslink reaction at room temperature. Subsequently, excess COL1 was washed off, and the COL1-coated gel was blocked in 1% ethanolamine in 50 mM HEPES for 30 min at 4 °C. Finally, the COL1-coated gels were rinsed using FBS-free culture medium and preserved in FBS-free culture medium at 4 °C overnight for usage. Approximately 3×10^6^ of HCC cells in 0.32 ml medium were spread onto a COL1-coated polyacrylamide gel with tunable stiffness for 2 h culture at room temperature, and then 3 ml culture medium were added into the attached cells for 48 h culture in culture incubator.

**Chemical reagents**

TGF β1, GW5074 and diphenylene-iodonium chloride (DPI) were obtained from Sigma-Aldrich (St Louis, USA). Transmembrane protein extraction kit was purchased from Merck Millipore (Boston, USA). Matrigel, collagen1, fibronectin and laminin were obtained from BD Biosciences (New Jersey, USA). Aspartate transaminase (AST), alanine transaminase (ALT), albumin (ALB), alkaline phosphatase (ALP), total bilirubin (TB), globuli (GLOB), γ-glutamyl transpeptadase (GGT) assay kits were from the Nanjing Jiancheng Bioengineering Institute (Nanjing, China). Antibodies against integrinβ1, integrinα5, FAK, E-cadherin, N-cadherin, vimentin, α-SMA, Snail, Smad2/3, p-Smad2(s465/467), p-Smad3(s423/425) , p-eIF4E (s209), Raf1, p-Raf1(s259), GAPDH were purchased from Cell Signal Technology (Boston, USA), and collagen I, LOX, eIF4E, S100A11, TGFβ1, Furin from Abcam (Cambridge, UK); Na+-K+-ATPase was from Proteintech (Chicago, USA). Antibodies against S100A11, HMGA2 were manufactured by Proteintech (Chicago, USA). Monoclonal antibodies against p67phox and p47phox was from BD Biosciences (New Jersey, USA) and Santa Cruz (Santa Cruz, USA).

**Stable knockdown expression of integrin β1 and integrin α5 in HCC cells with lentivirus vector**

The pGCSIL-shRNA-integrinβ1 lentivirus vectors and pGCSIL-shRNA-integrinα5 lentivirus vectors were constructed in collaboration with Shanghai Genechem, Co. Ltd. Briefly, shRNA for integrinβ1 (ITGβ1, 5’-CCTCCAGATGACATAGAAA-3’), integrinα5 (ITGα5, 5’-TCAGGAACGAGTCAGAATT-3’) and scramble(5’-TTCTCCGAACGTGTCACGT-3’) were synthesized and cloned into the plasmid pGCSIL, respectively. Target shRNA sequence in the recombinant plasmid was validated by PCR and sequencing analysis. Subsequently, 20 μg recombinant plasmid, 15 μg pHelper 1.0 plasmid and10 μg pHelper 2.0 plasmid were incubated in same volume Opti-MEM for 5 min at room temperature. The mixture was co-transfected into HEK293T cells by the help of lipofectamine 2000. The viral supernatant was harvested48h after transfection, and the viral titer of lentivirus (LV)-shRNA-vectors was determined. When HCC cells grew and reached 40% confluence in a 6-well plate, they were infected with LV-shRNA-ITGβ1 or LV-shRNA-ITGα5 or scramble plus ENi.S and 5μg/ml polybrene (MHCC97H cells with a multiplicity of infection (MOI) of 5, Hep3B cells with a multiplicity of infection (MOI) of 10).After 48h, the infected HCC cells were used for further experiment.

**Quantitative reverse transcription polymerase chain reaction (qRT-PCR)**

Total RNA from HCC cells was extracted using Trizol reagent (Invitrogen, Carlsbad, USA). The complementary DNA (cDNA) was synthesized using the Superscript First-Strand Synthesis System (Thermo Scientific, Waltham, USA), and cDNA template was used for gene amplification with a gene specific primer and SYBR Green PCR Master Mix kit (Invitrogen, Carlsbad, USA). Relative gene expression was normalized to GAPDH and reported as 2^- Δ Ct^[Δ Ct = Ct (MMP2 or other gene)-Ct (GAPDH)]. The primer sequences of genes are listed in the Supporting STable1.

**Western blot**

The procedure of western blot was same as the method described previously (You et al. Oncotarget. 2016;7(22):32221-31.). The diluted primary antibodies were as follows: integrinβ1, integrinα5, FAK, E-cadherin, N-cadherin, vimentin, α-SMA, Snail, Smad2/3, p-Smad2, p-Smad3, GAPDH, collagen 1, LOX, eIF4E, S100A11, p-eIF4E, Raf1, p-Raf1, gp91phox, Na+-K+-ATPase,HMGA2(1:1000), Furin(1:2000), TGFβ1(1:800), p67phox, p47phox (1:500) and HRP-conjugated secondary antibody (1:5000, Dingguo Bio, Beijing, China).

**Measurement of cell movement**

Cell movement was measured by the real-time cell monitoring system including a Cell-IQ cell culture platform (Chip-Man Technologies, Tampere, Finland), a phase-contrast microscope (Nikon CFI Achromat phase contrast objective with 10 × magnification) and a camera (Nikon, Tokoyo, Japan). Cell images were captured at 30 min intervals for 72 h. Time-lapse data was monitored and analyzed using a freely distributed Image software Cell-IQ Imagen v2.9.5c (McMaster Biophotonics Facility, Hamilton, Canada) combining with the Manual Tracking plug-in (Fabrice Cordelieres, Institut Curie, Orsay, France). The movement state of individual cell was analyzed in the image field by metering the distance of cell movement

**Immunofluorescence staining and Immunohistochemistry**

Cell slide was fixed in 4% paraformaldehyde in PBS, and the cells was permeabilized with PBS containing 0.5% Triton-X-100. F-actin in cells was stained using Alexa-488 Phalloidin (0.17μΜ, Invitrogen, Carlsbad, USA). Nuclear DNA was labelled with 4’, 6’-diamidino-2-phenyl-indole dihydrochloride (Dako, Glostrup, Denmark). The stained HCC cells were observed using confocal microscopy.

Immunohistochemical staining was performed as the method described previously (Dong et al. Biochem Biophys Res Commun. 2014 14;444(3):427-32). The diluted primary antibodies were as follows: E-cadherin, vimentin, TGFβ1 (1:100), Snail(1:150).

**Recombinant plasmid construction and transient transfection**

The pFU-GW-shRNA-S100A11 plasmid and pFU-GW-shRNA- eIF4E plasmid were constructed in collaboration with Shanghai Genechem, Co. Ltd. shRNA-S100A11(5'-TCCAGAAGTATGCTGGAAA-3'), shRNA-eIF4E(5'-GACTACAGAAGAGGAGAAA-3'), and scramble (5’-TTCTCCGAACGTGTCACGT-3’)were respectively synthesized and cloned into plasmid pFU-GW. shRNA sequence of target gene in the recombinant plasmid was confirmed by PCR and sequence analysis. S100A11-expressing plasmid (pEGFP-OE-S100A11) and eIF4E-expressing plasmid (pEGFP-OE-eIF4E)were also constructed in collaboration with Shanghai Genechem, Co. Ltd. Briefly, the cDNA encoding S100A11 and eIF4E were amplified by RT-PCR. The primer sequence of two target genes are as follows:S100A11 (F)TACCGGACTCAGATCTCGAGATGGCAAAAATCTCCAGC,S100A11(R)GATCCCGGGCCCGCGGTACCGTGGTCCGCTTCTGGGAAGG;eIF4E(F)TACCGGACTCAGATCTCGAGATGGCGACTGTCGAACCGeIF4E(R)GATCCCGGGCCCGCGGTACCGTAACAACAAACCTATTTTTAGTGGTGG

Subsequently, the synthesized target gene was cloned into pEGFPvector. Expression sequence of target gene in recombinant plasmid was confirmed by PCR and sequence analysis. HCC cells were transfected with recombinant expression plasmids using lipofectamine 2000 (Invitrogen, Carlsbad, USA) when they grew and reached 80% confluence.

**Detection of reactive oxygen species (ROS) product**

Intracellular ROS levels were determined by measuring the oxidative conversion of non-fluorescent 2’,7’-dichlorofluorescein (DCFH) to fluorescent dichlorofluorescein (DCF). Appropriate 1×10^6^HCC cells were incubated with 10 μmol/L2’,7’-dichlorofluorescein diacetate (DCFH-DA, KeyGen Biotech, Nanjing, China) at 37 °C for 30 min according to the manufacturer’ s instructions, and then washed twice with PBS. The cells were suspended in serum-free medium for detection of ROS accumulation. FACS Calibur flow cytometry system (BD, New Jersey, USA) was set as an excitation wavelength of 488 nm and an emission wavelength of 538 nm.

**Membrane protein extraction**

Membrane protein of HCC cells was extracted using Transmembrane Protein Extraction Kit. Brieﬂy, HCC cells grown on different stiffness substrates were collected, and then lysed in solution A (0.32M sucrose,5mM Tris-HCl (PH 7.5), 120mM KCl, 1mM EDTA, 0.2mM PMSF, 1ug/ml Leupeptin, 1ug/ml Pepstatin A, 1ug/m Aprotinin). Cell lysates were centrifuged at 14000 g for 30 min at 4° C. Cytoplasmic proteins were obtained from the supernatant. The precipitate was further treated with solution B (20mM HEPES(PH 7.5), 10% glycerol, 2% Triton X-100, 1mM EDTA, 0.2Mm PMSF, 1ug/ml Leupeptin, 1ug/ml Pepstatin A, 1ug/ml Aprotinin), then it was centrifuged at 14000 g for 5 min at 4 ° C. Membrane proteins were extracted from the supernatant and stored at 80 °C for further analysis.

**Co-immunoprecipitation (CO-IP)**

MHCC97H or Hep3B cells transfected with pEGFP-OE-S100A11 grown on different stiffness substrates were collected, and their total proteins were prepared using immunoprecipitation lysis buffer (20 mM Tris-HCl, pH 7.6; 150 mM NaCl; 1 mM EDTA; 0.5% NP-40; 10% glycerol; 1 mM PMSF; protease inhibitor cocktail). The extracted total protein was pretreated with normal IgG and protein A/G plus-agarose beads (Santa Cruz Biotechnology, Santa Cruz, CA, USA) at 4 °C for 2 hrs. Then their supernatants were incubated with IgG and S100A11 antibody together with protein A/G plus-agarose beads overnight at 4°C respectively. Subsequently, the collected beads were washed five times using washing buffer (50 mM Tris-HCl, pH 7.6; 300 mM NaCl; 1 mM EDTA; 0.5% NP-40; 10% glycerol). The eluant and post-elution beads were boiled in the loading buffer, resolved on SDS-PAGE. The whole-cell lysates and immunoprecipitation products were subject to immunoblotting to detect p67phox, p47phox and S100A11.

**Phosphoprotein profile changes in MHCC97H cells under different stiffness stimulation by the cancer signaling phosphorylation antibody array**

Briefly, MHCC97H cells grown on different stiffness substrate were collected. Cell lysates were biotinylated using Antibody Array Assay Kit (Full Moon Biosystems, Inc. Sunnyvale, CA, USA ). The antibody microarray slides were blocked in a blocking solution (3% normal goat serum (NGS), 1% bovine serum albumin (BSA), 0.1 mM Tris-buffered saline (TBS, pH 7.6)) for 30 min at room temperature, then it was rinsed with Milli-Q grade water for 3-5 min and dried in compressed nitrogen. Subsequently, the slides were incubated with the biotin-labeled cell lysates (100 μg protein) in coupling solution (2mM N,N′-dimethyl-p-phenylendiamine (DPP), 3.2 mM 4-chloro-1-naphthoyl) at room temperature for 2 h. The slides were washed 4-5 times with 1χWash Solution(120 mM KCl, 1.2 mM MgCl2 , 2.5 mM EDTA, 50 mM Tris-HCl (pH 7.4)) and rinsed extensively with Milli-Q grade water. Bound biotinylated proteins in the slides were reacted with Cy3-conjugated streptavidin. The slides were scanned using a GenePix 4000 scanner, and the images were analyzed with GenePix Pro 6.0 (Molecular Devices, Sunnyvale, CA, USA). The phosphorylation ratio was calculated as phosphorylation ratio = phospho value / unphospho value. Antibody array analysis was performed in collaboration with Wayen Biotechnology (Shanghai, China)

**miRNA extraction and qRT-PCR for miRNA expression**

miRNA in HCC cells was extracted according to manufacturer’s protocol (Majorbio, Shanghai) . All-in-One™ miRNA qRT-PCR Detection Kit (Genecopoeia, USA) was used for amplifying miRNA. miRNA 2ug, PolyA Polymerase(2.5U/ul)1ul, RTase Mix1ul, 5×Reaction Buffer5ul and ddH₂O were mixed to a final volume of 25ul, and incubated at 37 ℃ for 60 min, and then 85 ℃ for 5 min. Products were diluted 5 times for downstream qPCR detection. All-in-One qPCR Mix 10ul, miRNA-24-3p primer or U6 primer (reference primer) 2ul, Universal Adaptor PCR Primer 2ul, First strand cDNA 2ul and 4ul ddH_2_O were mixed to a final volume of 20ul, and denatured at 95 ℃ for 10 min, followed by 40 cycles of amplification (denaturation 95, 10 sec; annealing 60, 20 sec; extension 72, 10 sec) for qPCR detection. miR-24-3p primer and U6 primer sequence were purchased from FulenGen Co, Guangzhou, China.

**Construction of lentivirus-mediated miRNA-24-3p overexpression and interference**

miR-24-3p interference sequence(CCGGCTGTTCCTGCTGAACTGAGCCATTTTTG) was cloned into GV280 vector to construct recombinant plasmid, and miR-24-3p overexpression sequence(TCCAGGCCTTCGCGTCTCCTGCGCCAGCAGACGGTGCCCACGGAGCTCCCAGCTGAGGCGCTGCTTCTCCGGGCTGTCGATTGGACCCGCCCTCCGGTGCCTACTGAGCTGATATCAGTTCTCATTTTACACACTGGCTCAGTTCAGCAGGAACAGGAGTCGAGCCCTTGAGCAAAAAGCCTTCGTGTCTGTAAGTGCCCGAGGCTCAGGAGAGCTGGGGCTCCCACTCGCGGCAGACAGGCC) was cloned into GV369 vector to construct recombinant plasmid. Recombinant plasmid was transformed into competent E.coli, and the positive clones were identified by PCR and sequence analysis. The selected positive clone was amplified and the plasmids were extracted. Above plasmids and packing plasmids Helper1.0 and Helper2.0 were co-transfected into 293T cells for obtainingLV-miR-24-3p-OE and LV-miR-24-3p-shRNA. The amount of the used lentivirus was calculated according to cell number and MOI value (MHCC97H: 5, Hep3B: 10). Transfection solution was prepared by mixing 800ul culture medium, 200ul enhanced Solution (Genechem, Shanghai) and 1ul polybrene (Genechem, Shanghai). The transfection solution was replaced with fresh culture medium after transfection for 12 hours. LV-miR-24-3p-OE and LV-miR-24-3p-shRNA were prepared in collaboration with Genechem Co. (Shanghai, China).

**Dual luciferase Assay**

Dual luciferase assay was performed according to manufacturer’s protocol. Wildtype FURIN 3’UTR or mutant FURINR 3’UTR was cloned into pMIR-REPORT plasmid using restriction endonuclease Mlu I and Hind III. Recombinant plasmids were transfected into 293T cells using Lipofectamine 2000. Simultaneously, miRNA-24-3p and negative control were introduced into cells. Dual-Luciferase® Reporter Assay System (Promega, USA) was used to evaluate fluorescence intensity. For 96-well plate, 50ul 1χPLB was dispensed to each culture vessel for passive lysis for 15 min at room temperature. Firefly luciferase activity was detected using 100ul LARII by luminometer. Subsequently, renilla luciferase activity was detected using 100µl of Stop&Glo® Reagent. This experiment was done in collaboration with Obio Technology Corp.,Ltd (Shanghai, China).

**Enzyme-linked immunosorbent assay (ELISA)**

The levels of TGF β1 in culture supernatants of HCC cells grown on different stiffness substrates were measured by ELISA. Approximately 3×106 of HCC cells were seeded onto a COL1-coated polyacrylamide gel with tunable stiffness for 48 h culture, and then their culture supernatants were collected. The levels of TGF β1 in culture supernatant were measured by ELISA (Boster Biological Technology, Wuhan, China). The procedures of ELISA were performed according to manufacturer’s instructions.
