# Supplementary figures and images for: Higher matrix stiffness as an independent initiator triggers epithelial-mesenchymal transition and facilitates HCC metastasis
Source: J Hematol Oncol. 2019 Nov 8;12:112. doi: 10.1186/s13045-019-0795-5 (PMC6839087; doi:10.1186/s13045-019-0795-5)

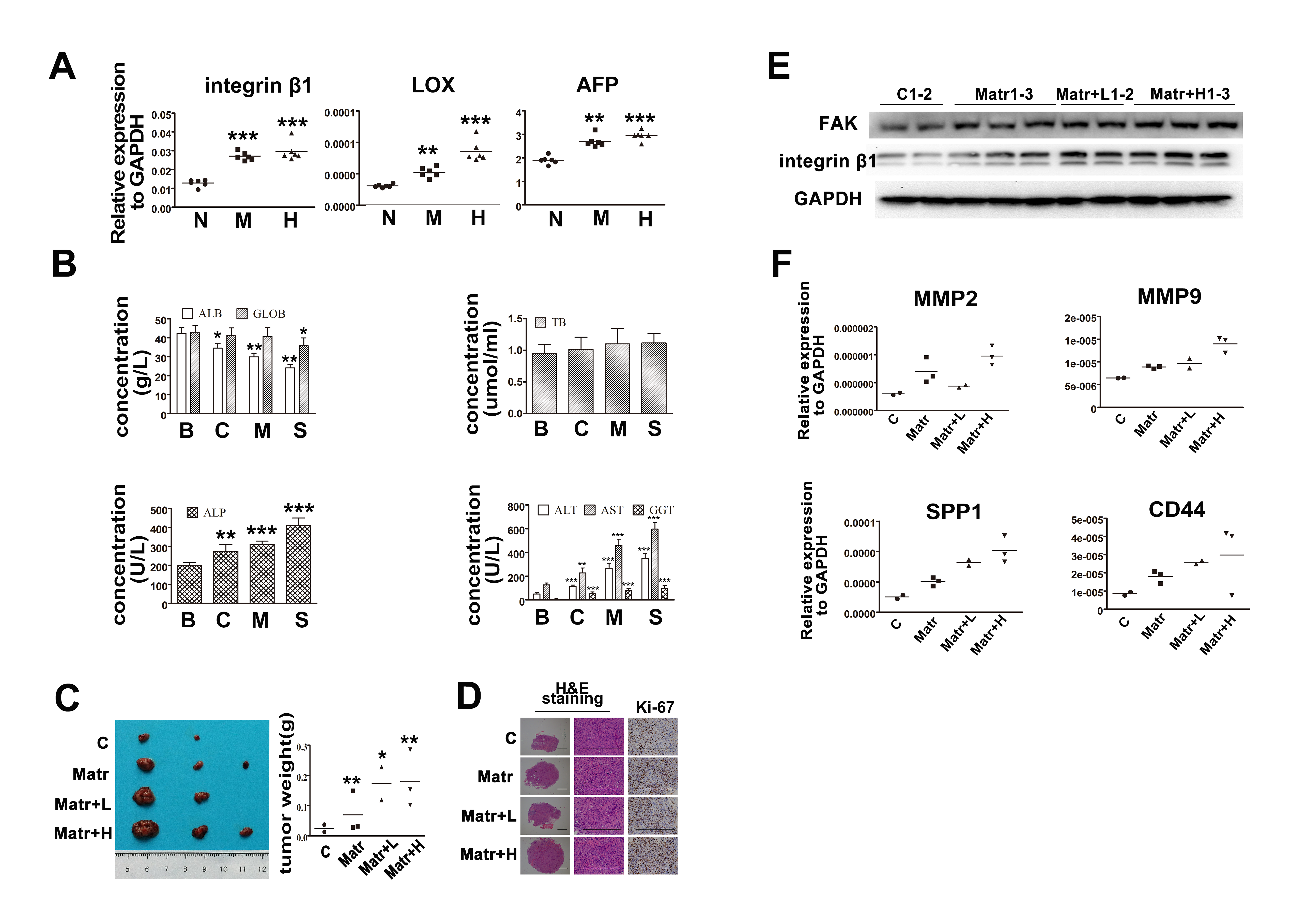

Supplement: Supplementary file 2 — Additional file 2: Figure S1. Higher liver stiffness promotes the growth of HCC and facilitates HCC invasion and metastasis in vivo. (A) The mRNA expression of integrin β1, LOX and AFP in orthotopic HCC tumors in groups N, M and H. (B) Liver function analysis of buffalo rat HCC models with different liver stiffness backgrounds. B, Healthy buffalo rats, C, HCC buffalo rats with normal liver stiffness, M, HCC buffalo rats with medium liver stiffness, S, HCC buffalo rats with high liver stiffness. (C) Gross appearance and wet weight of subcutaneous tumors derived from Hep3B cells mixed in Matrigel and varied concentration of COL1. (D) Histochemistry analysis of subcutaneous tumors and their Ki-67 expression. (E) The expressions of integrin β1 and FAK in subcutaneous tumors. (F) The expressions of MMP2, MMP9, SPP1, CD44 in subcutaneous tumors. C, Hep3B cells; Matr., Hep3B cells in Matrigel; Matr+L., Hep3B cells in Matrigel and low concentration COL1; Matr+H., Hep3B cells in Matrigel and high concentration COL1. [file 13045_2019_795_MOESM2_ESM.tif]

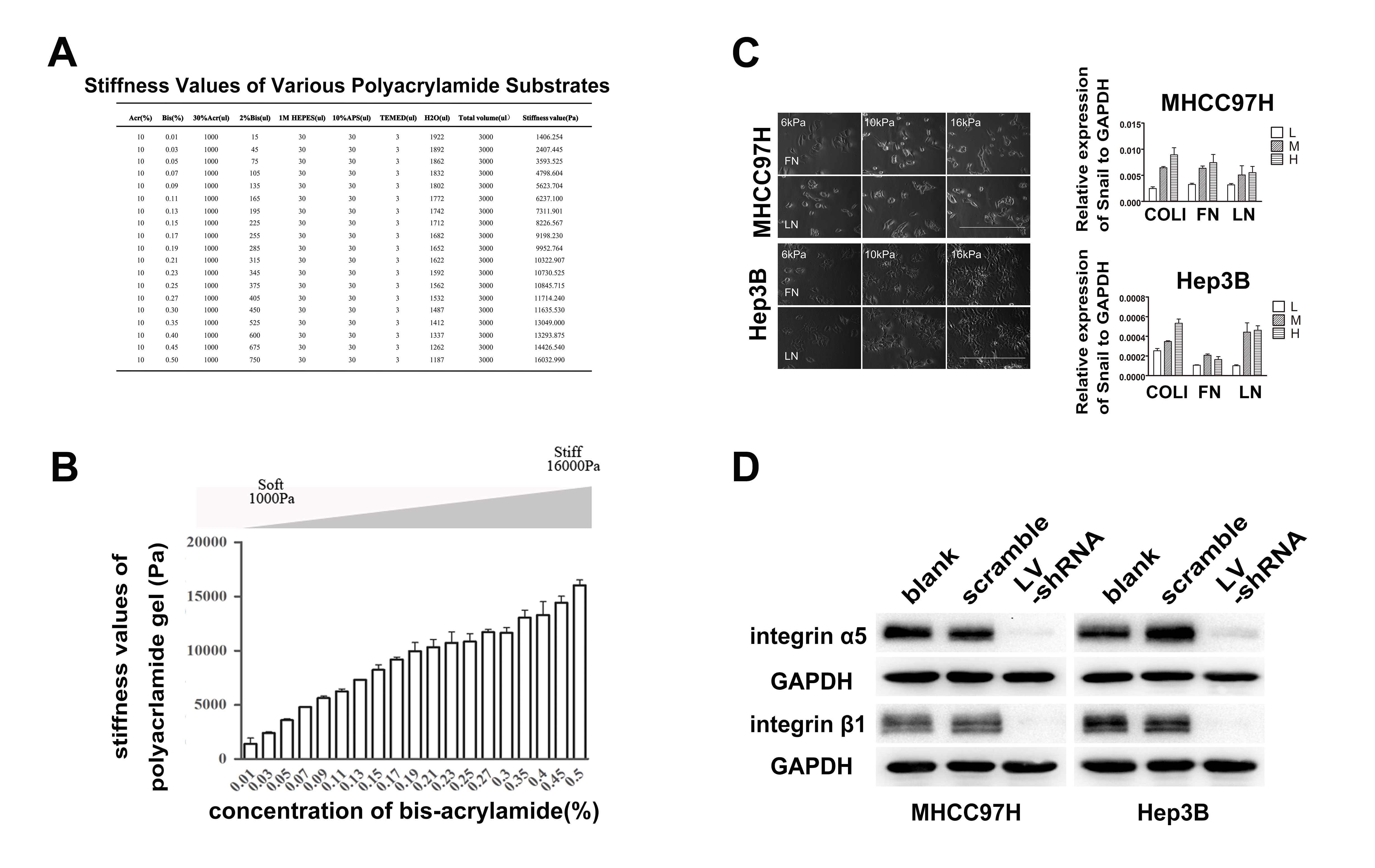

Supplement: Supplementary file 3 — Additional file 3: Figure S2. An in vitro system of COL1-coated polyacrylamide gels with tunable stiffness. (A) Preparation of an in vitro system of COL1-coated polyacrylamide gels with tunable stiffness (B) Stiffness values of different stiffness substrates (C) Morphology alteration of HCC cells grown on FN/LN- coated gels with tunable stiffness and their Snail expression. (D) Expressions of integrin α5 or integrin β1 in HCC cells transfected with LV-shRNA-ITG α5 and LV-shRNA-ITG β1. [file 13045_2019_795_MOESM3_ESM.tif]

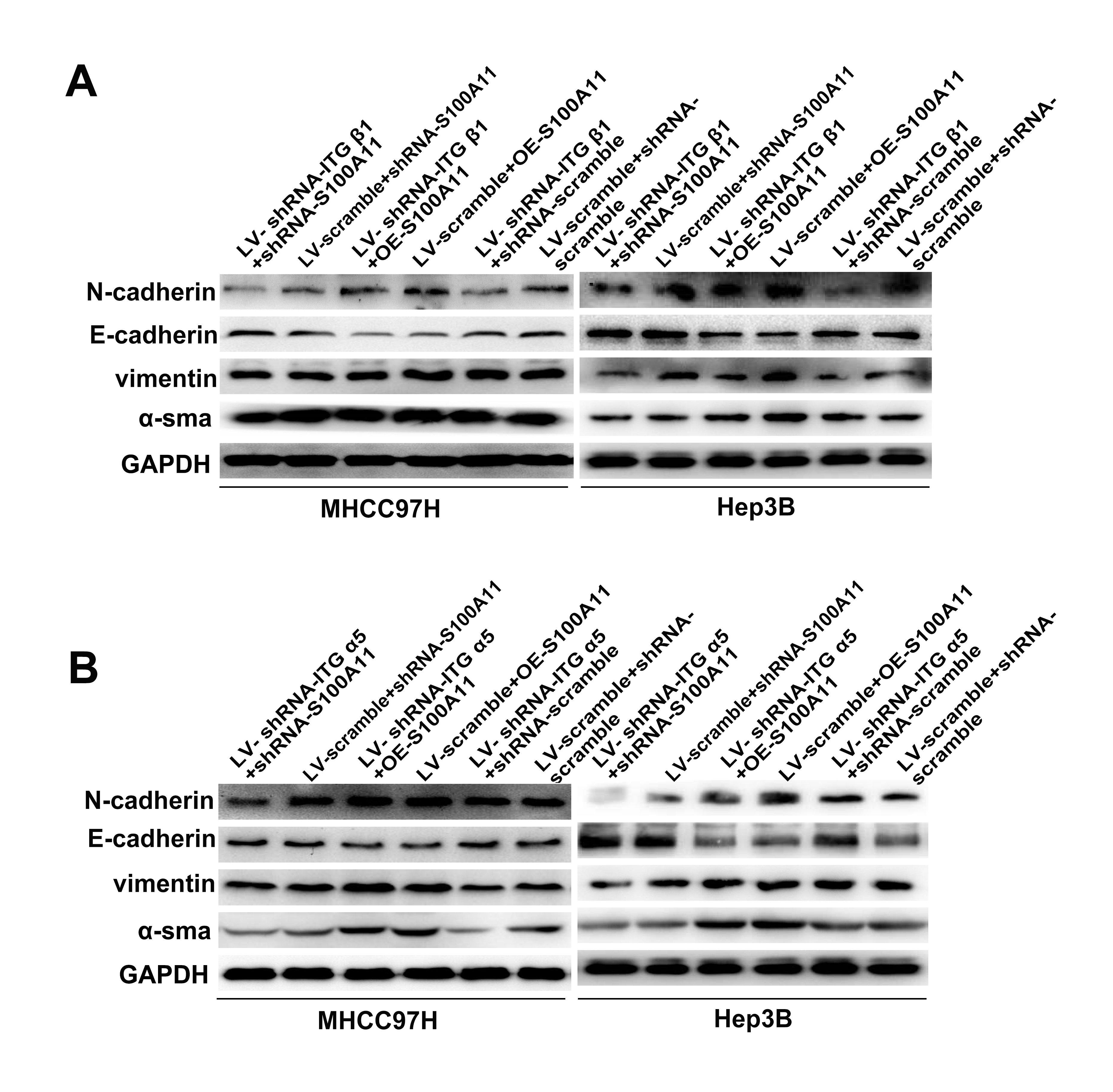

Supplement: Supplementary file 4 — Additional file 4: Figure S3. S100A11 participates in stiffness-induced EMT in HCC cells. (A) The expression of EMT markers in HCC cells co-transfected with pFU-GW-shRNA-S100A11 and LV-shRNA-ITGβ1 or pEGFP-OE-S100A11 and LV-shRNA-ITGβ1 under higher stiffness stimulation. (B) The expression of EMT markers in HCC cells co-transfected with pFU-GW-shRNA-S100A11 and LV-shRNA-ITGα5 or pEGFP-OE-S100A11 and LV-shRNA-ITGα5 under higher stiffness stimulation. [file 13045_2019_795_MOESM4_ESM.tif]

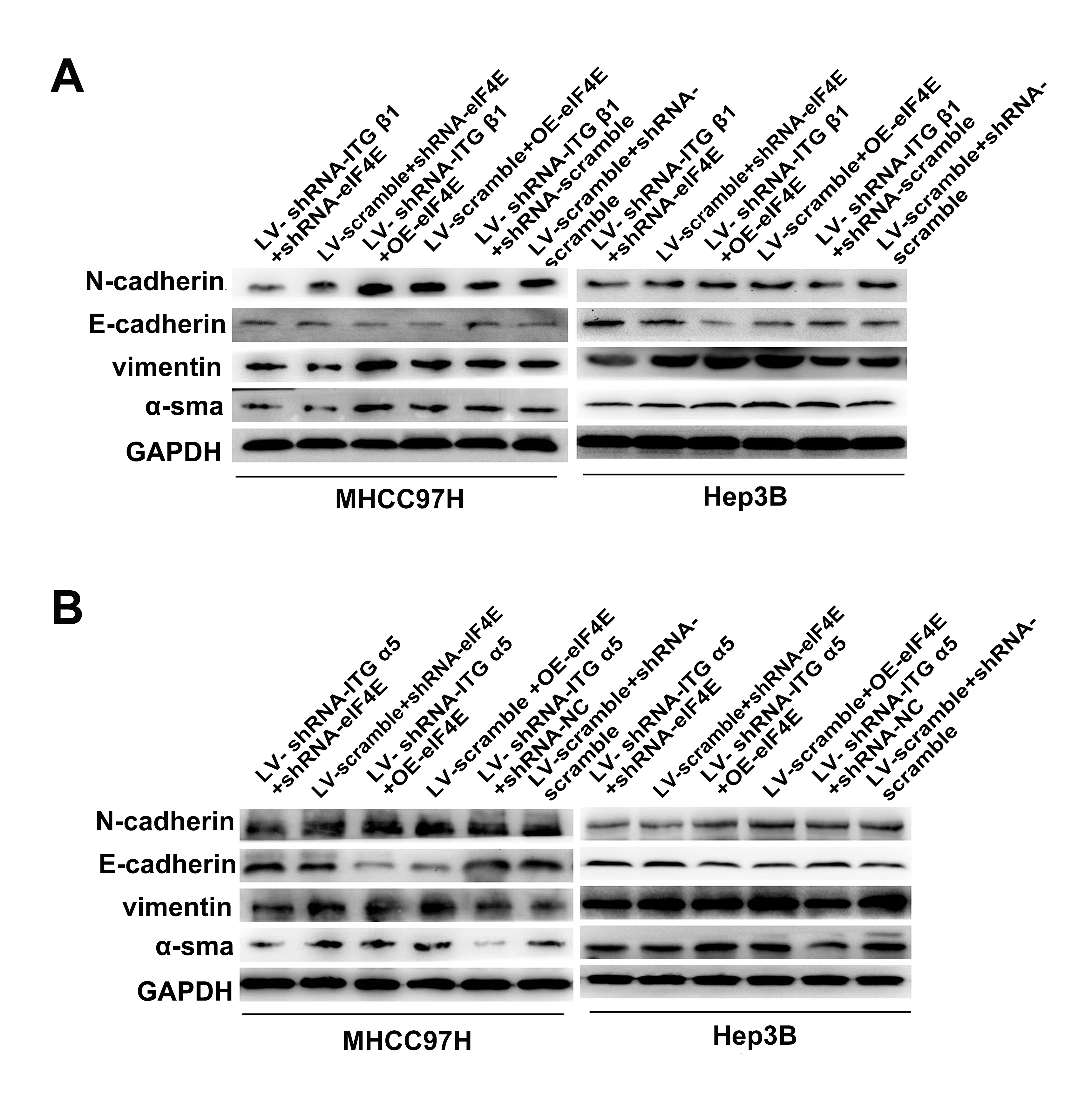

Supplement: Supplementary file 5 — Additional file 5: Figure S4. eIF4E participates in stiffness-induced EMT in HCC cells. (A) The expression of EMT markers in HCC cells co-transfected with pFU-GW-shRNA- eIF4E and LV-shRNA-ITGα5 or pEGFP-OE-eIF4E overexpression and LV-shRNA-ITGα5 under higher stiffness stimulation (B) The expression of EMT markers in HCC cells co-transfected with pFU-GW-shRNA- eIF4E and LV-shRNA-ITGα5 or pEGFP-OE-eIF4E and LV-shRNA-ITGα5 under higher stiffness stimulation. [file 13045_2019_795_MOESM5_ESM.tif]

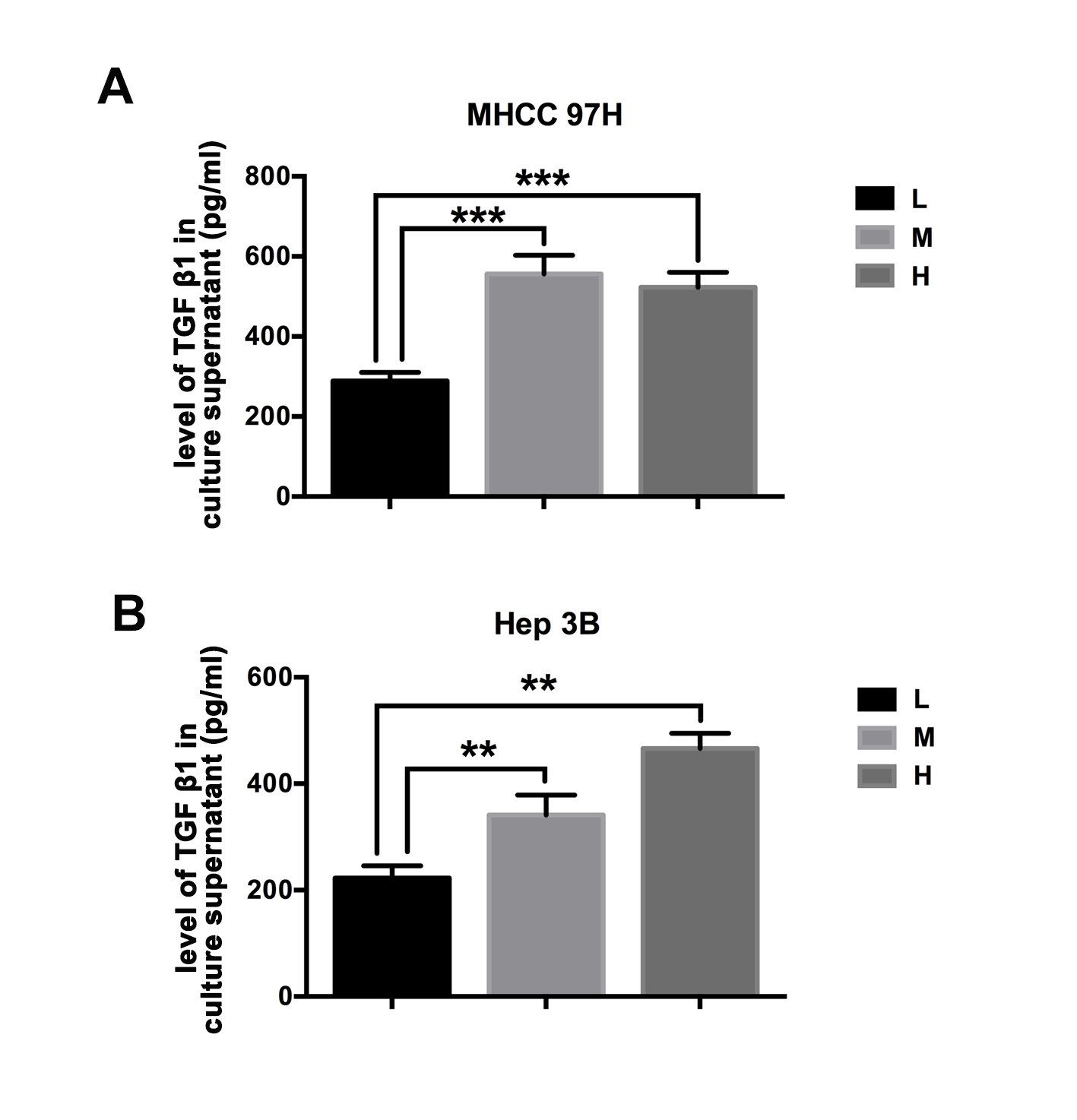

Supplement: Supplementary file 6 — Additional file 6: Figure S5. The levels of TGF-β1 in culture supernatants of HCC cells grown on different stiffness substrates. [file 13045_2019_795_MOESM6_ESM.tif]
