# Supplementary material for: Higher matrix stiffness as an independent initiator triggers epithelial-mesenchymal transition and facilitates HCC metastasis
Source: J Hematol Oncol. 2019 Nov 8;12:112. doi: 10.1186/s13045-019-0795-5 (PMC6839087; doi:10.1186/s13045-019-0795-5)
Supplement: Supplementary file 7 — Additional file 7: Table S1. Primer pairs used for qRT-PCR [file 13045_2019_795_MOESM7_ESM.docx]

**Table S1.** **Primer pairs used for qRT-PCR**

| Gene symbol | Sequence |
| --- | --- |
| MMP9(Rat)  MMP2(Rat)  SPP1(Rat)  CD44(Rat)  AFP (Rat)  LOX (Rat)  Integrinβ1(Rat)  GAPDH(Rat)  MMP9(Human)  MMP2(Human)  SPP1(Human)  CD44(Human)  GAPDH(Human) | Forward: 5'- CCGAGACCTGAAAACC - 3'  Reverse: 5'- GGCTGCCCGAGTGTAA - 3'  Forward: 5'- GACCCACGCCTACACT - 3'  Reverse: 5'- CGCCAAATAAACCGA - 3'  Forward: 5'- CCCGATGCCACAGAT - 3'  Reverse: 5'- GCACGCTCAGACGCT - 3'  Forward: 5'- TCTGATTCTTGCCGTCTG - 3'  Reverse: 5'- TTCACTTGGTTTCCTGTC - 3'  Forward: 5'- TGGAGTGCCTACAGGATGG - 3'  Reverse: 5'- TCGCTTGGGTTCAGAGTTA - 3'  Forward: 5'- GGACTCCAAGCCCATC - 3'  Reverse: 5'-CGGCGAGAAACCAACT - 3'  Forward: 5'- AATGGACGAAAGTGCTCTAA- 3'  Reverse: 5'- AACTGAAGGACCACCTCTAC - 3'  Forward: 5'- GCCTTCCGTGTTCCTA - 3'  Reverse: 5'- AGACAACCTGGTCCTCA- 3'  Forward: 5'- CTTTGGACACGCACGAC - 3'  Reverse: 5'- CCACCTGGTTCAACTCACT - 3'  Forward: 5'- GTTCATTTGGCGGACTGT - 3'  Reverse: 5'- AGGGTGCTGGCTGAGTAG - 3'  Forward: 5'-CAGTGATTTGCTTTTGCC- 3'  Reverse: 5'-AGATGGGTCAGGGTTTAG- 3'  Forward: 5'- GGTGAACAAGGAGTCGTC - 3'  Reverse: 5'- TTCCAAGATAATGGTGTAGGTG - 3'  Forward: 5'- CTCCTCCACCTTTGACGC - 3'  Reverse: 5'- CCACCACCCTGTTGCTGT- 3' |
