# Supplementary material for: Higher matrix stiffness as an independent initiator triggers epithelial-mesenchymal transition and facilitates HCC metastasis
Source: J Hematol Oncol. 2019 Nov 8;12:112. doi: 10.1186/s13045-019-0795-5 (PMC6839087; doi:10.1186/s13045-019-0795-5)
Supplement: Supplementary file 8 — Additional file 8: Table S2. Different phosphorylation signal proteins in expression pattern of “a”. Table S3. Different phosphorylation signal proteins in expression pattern of“b”. Table S4. Different phosphorylation signal proteins in expression pattern of “c”. Table S5. Different phosphorylation signal proteins in expression pattern of “d”. [file 13045_2019_795_MOESM8_ESM.docx]

**Table S2. Different phosphorylation signal proteins in expression pattern of “a”**

| Name | M/L | H/L |
| --- | --- | --- |
| Akt2 (Phospho-Ser474) | 1.341159 | 1.341042 |
| BAD (Phospho-Ser112) | 1.221586 | 1.251318 |
| BAD (Phospho-Ser155) | 1.280852 | 1.346228 |
| Caveolin-1 (Phospho-Tyr14) | 1.068973 | 1.135016 |
| Chk2 (Phospho-Thr68) | 1.047955 | 1.199418 |
| c-Jun (Phospho-Thr239) | 1.167389 | 1.247785 |
| Elk-1 (Phospho-Ser383) | 1.393181 | 2.230209 |
| FKHR (Phospho-Ser256) | 1.234872 | 1.2762 |
| HDAC8 (Phospho-Ser39) | 1.158201 | 1.163009 |
| IKK alpha (Phospho-Thr23) | 1.105983 | 1.213709 |
| JAK2 (Phospho-Tyr1007) | 1.098454 | 1.379226 |
| JunB (Phospho-Ser79) | 1.106569 | 1.33804 |
| JunD (Phospho-Ser255) | 1.351233 | 1.67398 |
| MSK1 (Phospho-Ser376) | 1.081255 | 1.368777 |
| Myc (Phospho-Ser373) | 1.28415 | 1.321949 |
| Myc (Phospho-Ser62) | 1.067625 | 1.097201 |
| Myc (Phospho-Thr358) | 1.079311 | 1.117626 |
| Myc (Phospho-Thr58) | 1.237683 | 1.497363 |
| NFkB-p100/p52 (Phospho-Ser869) | 1.638938 | 1.839713 |
| NFkB-p105/p50 (Phospho-Ser893) | 1.331049 | 1.420086 |
| NFkB-p65 (Phospho-Ser529) | 1.079802 | 1.114654 |
| p21Cip1 (Phospho-Thr145) | 1.0714 | 1.147453 |
| p44/42 MAP Kinase (Phospho-Tyr204) | 1.065052 | 1.203619 |
| p53 (Phospho-Ser6) | 1.145695 | 1.268244 |
| PI3-kinase p85-subunit alpha/gamma (Phospho-Tyr467/Tyr199) | 1.013151 | 1.035911 |
| Pyk2 (Phospho-Tyr402) | 1.16289 | 1.244007 |
| Rac1/cdc42 (Phospho-Ser71) | 1.038099 | 1.504115 |
| Src (Phospho-Tyr529) | 1.200703 | 1.350585 |
| Tau (Phospho-Ser404) | 1.01027 | 1.078196 |

**Table S3. Different phosphorylation signal proteins in expression pattern of“b”**

| Name | M/L | H/L |
| --- | --- | --- |
| Raf1 (Phospho-Ser259) | 2.503118 | 2.372118 |
| GSK3 beta (Phospho-Ser9) | 1.97319 | 1.939114 |
| STAT6 (Phospho-Tyr641) | 1.842692 | 1.354126 |
| BCL-2 (Phospho-Ser70) | 1.841408 | 1.694976 |
| STAT5A (Phospho-Ser780) | 1.832953 | 1.631593 |
| JunB (Phospho-Ser259) | 1.791544 | 1.621338 |
| STAT5A (Phospho-Tyr694) | 1.764535 | 1.31243 |
| Rel (Phospho-Ser503) | 1.739444 | 1.520269 |
| CREB (Phospho-Ser133) | 1.652706 | 1.238253 |
| Akt (Phospho-Ser473) | 1.626328 | 1.232197 |
| Akt (Phospho-Thr308) | 1.610202 | 1.055302 |
| STAT3 (Phospho-Ser727) | 1.607702 | 1.361321 |
| STAT4 (Phospho-Tyr693) | 1.602048 | 1.285229 |
| c-Jun (Phospho-Ser73) | 1.586418 | 1.223816 |
| STAT6 (Phospho-Thr645) | 1.549928 | 1.219695 |
| NFkB-p100/p52 (Phospho-Ser865) | 1.541693 | 1.350208 |
| mTOR (Phospho-Ser2448) | 1.52838 | 1.032799 |
| Chk1 (Phospho-Ser345) | 1.522295 | 1.055536 |
| cdc25C (Phospho-Ser216) | 1.49409 | 1.129469 |
| GSK3 alpha (Phospho-Ser21) | 1.490077 | 1.267966 |
| c-Kit (Phospho-Tyr721) | 1.479961 | 1.276496 |
| PTEN (Phospho-Ser380/Phospho-Thr382/Phospho-Thr383) | 1.470363 | 1.159016 |
| Chk1 (Phospho-Ser280) | 1.460459 | 1.258742 |
| BRCA1 (Phospho-Ser1524) | 1.457993 | 1.146918 |
| Shc (Phospho-Tyr349) | 1.45002 | 0.844151 |
| NFkB-p65 (Phospho-Thr254) | 1.446176 | 1.25851 |
| 4E-BP1 (Phospho-Thr36) | 1.391513 | 1.199809 |
| FAK (Phospho-Tyr925) | 1.383928 | 1.108731 |
| FAK (Phospho-Tyr861) | 1.376762 | 1.356065 |
| Met (Phospho-Tyr1349) | 1.364774 | 0.985515 |
| elF4E (Phospho-Ser209) | 1.335145 | 1.057798 |
| SHP-2 (Phospho-Tyr580) | 1.318502 | 1.030184 |
| PDK1 (Phospho-Ser241) | 1.298804 | 0.425539 |
| BCL-2 (Phospho-Thr56) | 1.297899 | 1.259206 |
| Rb (Phospho-Ser780) | 1.297129 | 1.016751 |
| STAT1 (Phospho-Tyr701) | 1.295961 | 1.195391 |
| STAT3 (Phospho-Tyr705) | 1.294337 | 1.164243 |
| MKK3 (Phospho-Ser189) | 1.292454 | 1.074846 |
| PDGF Receptor beta (Phospho-Tyr751) | 1.292327 | 0.992621 |
| JAK2 (Phospho-Tyr221) | 1.289463 | 1.235429 |
| TYK2 (Phospho-Tyr1054) | 1.250936 | 1.156767 |
| HSP27 (Phospho-Ser15) | 1.250491 | 1.220498 |
| Chk1 (Phospho-Ser317) | 1.250477 | 0.90445 |
| NFkB-p105/p50 (Phospho-Ser907) | 1.208769 | 0.891572 |
| JAK1 (Phospho-Tyr1022) | 1.190231 | 1.026293 |
| BAD (Phospho-Ser136) | 1.189009 | 1.149916 |
| FGF Receptor 1 (Phospho-Tyr154) | 1.17732 | 1.030945 |
| Trk B (Phospho-Tyr515) | 1.177241 | 1.005228 |
| Integrin beta3 (Phospho-Tyr773) | 1.166227 | 1.028617 |
| Estrogen Receptor-alpha (Phospho-Ser167) | 1.151739 | 1.128058 |
| Fak (Phospho-Tyr397) | 1.150357 | 1.001323 |
| CDC2 (Phospho-Tyr15) | 1.149844 | 1.096862 |
| p27Kip1 (Phospho-Thr187) | 1.147427 | 1.108603 |
| Beta-Catenin (Phospho-Ser37) | 1.146939 | 1.111798 |
| Caspase 9 (Phospho-Ser196) | 1.13964 | 0.861957 |
| Keratin 18 (Phospho-Ser33) | 1.134399 | 1.001876 |
| IGF-1R (Phospho-Tyr1161) | 1.133541 | 1.100798 |
| Src (Phospho-Tyr418) | 1.118586 | 1.095807 |
| p27Kip1 (Phospho-Ser10) | 1.112953 | 0.885269 |
| eEF2K (Phospho-Ser366) | 1.103419 | 0.890364 |
| Caspase 9 (Phospho-Thr125) | 1.102462 | 0.82119 |
| Caspase 9 (Phospho-Tyr153) | 1.094835 | 1.079258 |
| Caspase-3 (Phospho-Ser150) | 1.088335 | 0.903645 |
| ICAM-1 (Phospho-Tyr512) | 1.068816 | 1.062551 |
| SAPK/JNK (Phospho-Thr183) | 1.05489 | 0.915742 |
| ERK3 (Phospho-Ser189) | 1.048499 | 0.985309 |
| CrkII (Phospho-Tyr221) | 1.046929 | 0.944278 |
| p44/42 MAP Kinase (Phospho-Thr202) | 1.03527 | 0.953606 |
| HSF1 (Phospho-Ser303) | 1.02032 | 0.880135 |
| IkB-a (Phospho-Ser32/Phospho-Ser36) | 1.013528 | 0.890568 |

**Table S4. Different phosphorylation signal proteins in expression pattern of “c”**

| Name | M/L | H/L |
| --- | --- | --- |
| HSP27 (Phospho-Ser78) | 0.937632 | 1.132414 |
| VEGFR2 (Phospho-Tyr951) | 0.937491 | 1.078945 |
| IkB-a (Phospho-Tyr42) | 0.919928 | 1.296618 |
| Integrin beta3 (Phospho-Tyr785) | 0.919316 | 1.255657 |
| HER2 (Phospho-Tyr877) | 0.915918 | 0.985304 |
| MEK1 (Phospho-Ser217) | 0.880484 | 1.225394 |
| Chk2 (Phospho-Ser516) | 0.872955 | 0.908841 |
| CDK2 (Phospho-Thr160) | 0.869059 | 0.949087 |
| ERK8 (Phospho-Thr175/Tyr177) | 0.867737 | 0.953856 |
| p53 (Phospho-Ser315) | 0.855515 | 0.979924 |
| c-Jun (Phospho-Ser243) | 0.842767 | 1.334672 |
| Beta-Catenin (Phospho-Thr41/Phospho-Ser45) | 0.842666 | 1.01576 |
| eIF2a (Phospho-Ser51) | 0.814046 | 1.138768 |
| PI3-kinase p85-alpha (Phospho-Tyr607) | 0.768338 | 1.110246 |
| HSP90B (Phospho-Ser254) | 0.766129 | 1.033973 |
| IkB-e (Phospho-Ser22) | 0.760824 | 1.006919 |
| BCL-XL (Phospho-Ser62) | 0.741127 | 2.272546 |
| MEK1 (Phospho-Thr291) | 0.714338 | 0.932732 |
| Histone H2A.X (Phospho-Ser139) | 0.698013 | 1.229049 |
| AMPK1 (Phospho-Thr174) | 0.69488 | 1.118703 |
| CaMKII (Phospho-Thr286) | 0.649742 | 0.924939 |
| STAT1 (Phospho-Ser727) | 0.617422 | 1.095218 |
| p70 S6 Kinase (Phospho-Ser424) | 0.598315 | 1.020043 |
| 14-3-3 zeta (Phospho-Ser58) | 0.552029 | 1.028573 |

**Table S5. Different phosphorylation signal proteins in expression pattern of “d”**

| Name | M/L | H/L |
| --- | --- | --- |
| BRCA1 (Phospho-Ser1423) | 0.776506 | 0.75187 |
| Caspase 9 (Phospho-Ser144) | 0.963313 | 0.936393 |
| cdc25A (Phospho-Ser75) | 0.87269 | 0.783281 |
| EGFR (Phospho-Tyr1110) | 0.902662 | 0.799162 |
| MDM2 (Phospho-Ser166) | 0.84005 | 0.803944 |
| MEK1 (Phospho-Ser221) | 0.786187 | 0.754344 |
| MEK-2 (Phospho-Thr394) | 0.770938 | 0.750237 |
| P38 MAPK (Phospho-Tyr182) | 0.641069 | 0.595637 |
